# Peptide Variant Detection by a Living Yeast Biosensor via an Epitope-Selective Protease

**DOI:** 10.34133/bdr.0003

**Published:** 2023-03-15

**Authors:** Tea Crnković, Benjamin J. Bokor, Mead E. Lockwood, Virginia W. Cornish

**Affiliations:** ^1^ Department of Chemistry, Columbia University, New York, NY 10027, USA.; ^2^ Department of Biological Sciences, Columbia University, New York, NY 10027, USA.; ^3^ School of General Studies, Columbia University, New York, NY 10027, USA.; ^4^ Department of Systems Biology, Columbia University, New York, NY 10027, USA.

## Abstract

We previously demonstrated that we could hijack the fungal pheromone signaling pathway to provide a living yeast biosensor where peptide biomarkers were recognized by G-protein-coupled receptors and engineered to transcribe a readout. Here, we demonstrated that the protease could be reintroduced to the biosensor to provide a simple mechanism for distinguishing single-amino-acid changes in peptide ligands that, otherwise, would likely be difficult to detect using binding-based assays. We characterized the dose–response curves for five fungal pheromone G-protein-coupled receptors, peptides, and proteases*—Saccharomyces cerevisiae*, *Candida albicans*, *Schizosaccharomyces pombe*, *Schizosaccharomyces octosporus*, and *Schizosaccharomyces japonicus*. Alanine scanning was carried out for the most selective of these—*S. cerevisiae* and *C. albicans*—with and without the protease. Two peptide variants were discovered, which showed diminished cleavage by the protease (CaPep2A and CaPep2A13A). Those peptides were then distinguished by utilizing the biosensor strains with and without the protease, which selectively cleaved and altered the apparent concentration of peptide required for half-maximal activation for 2 peptides—CaPep and CaPep13A, respectively—by more than one order of magnitude. These results support the hypothesis that the living yeast biosensor with a sequence-specific protease can translate single-amino-acid changes into more than one order of magnitude apparent shift in the concentration of peptide required for half-maximal activation. With further engineering by computational modeling and directed evolution, the biosensor could likely distinguish a wide variety of peptide sequences beyond the alanine scanning carried out here. In the future, we envision incorporating proteases into our living yeast biosensor for use as a point of care diagnostic, a scalable communication language, and other applications.

## Introduction

Mating in fungi is regulated by transcription factors encoded at the mating-type (MAT) loci. The most well-studied mating pathway is of *Saccharomyces cerevisiae* where haploid cells can be either a or α mating type (MATa and MATα, respectively) which, under appropriate conditions, can form diploid cells MATa/MATα [1]. Important molecules that are involved in the mating initiation and progress are pheromones, and *S. cerevisiae* of MATα secretes α-pheromone, a 13-amino-acid peptide, which is recognized by the Ste2 G-protein-coupled receptor (GPCR) on the surface of MATa cells [2]. α-Pheromone activation of the GPCR initiates a signal transduction cascade that includes the mitogen-activated protein kinase pathway, which initiates transcription of mating-associated genes that ultimately lead to cell fusion of 2 haploid cells into a diploid cell [3]. Another important component in yeast mating is the α-pheromone-specific aspartyl protease Bar1 secreted by MATa cells. The protease creates the α-pheromone gradient in the environment, which helps guide the mating partners, and it also aids in cell cycle arrest recovery if the mating does not occur [3].

In 2017, we reported a living yeast biosensor engineered with a modular cell surface receptor and a readout visible to the naked eye [4]. This biosensor is based on rewiring of yeast’s natural pheromone-responsive intracellular mating pathway. For the biosensor, we used the laboratory strain *S. cerevisiae* and modular fungal GPCRs that naturally respond to their cognate pheromone peptides, which can be engineered with directed evolution. In addition, for the readout, GPCR activation of the pheromone-responsive signaling pathway was engineered to activate transcription of the lycopene biosynthetic pathway. Lycopene is the pigment that turns tomatoes and other fruits red and, hence, requires no exogenous reagents or specialized equipment and gives a colorimetric readout visible to the naked eye.

**Fig. 1.**
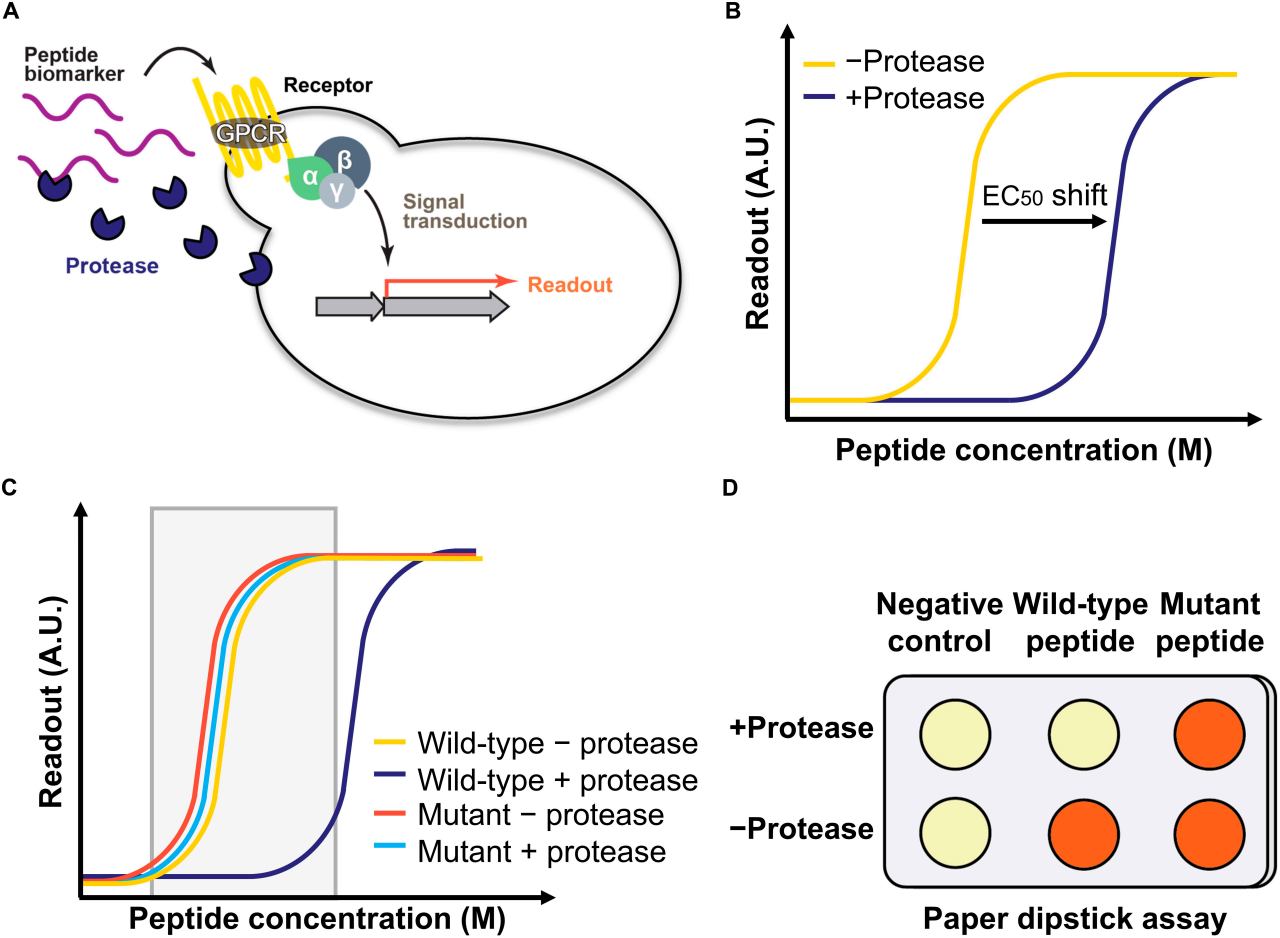
Design of protease-based live yeast biosensor. (A) Engineered yeast has a GPCR that induces readout expression in response to cognate peptide. In addition, a peptide protease is constitutively secreted by engineered yeast to cleave the cognate peptide. (B) Peptide dose–response plot illustrating the expected effect of the secreted protease. Peptide cleavage by the protease reduces GPCR activation as observed by the apparent shift in EC_50_. (C) Dose–response curve characterizing the readout for distinction of 2 peptide variants within expected peptide concentration range (gray box). Without protease, the GPCR response to both peptides should show similar activation curve. With protease, only one peptide is cleaved and will have shifted apparent EC_50_ outside gray box. (D) Illustration of a paper-based dipstick assay with engineered yeast patches responding to peptides differing in single-amino-acid (columns). The rows contain yeast expressing (+protease) or not expressing (−protease) the peptide protease. The orange pigment represents an inducible readout that can distinguish peptide variants. A.U., arbitrary units.

One component that was omitted for simplicity from the 2017 biosensor was the protease. We envisioned that we could reintroduce the protease for enhanced and more precise biosensing capabilities of our initial biosensor (Fig. 1A). We hypothesized that the protease could provide a simple mechanism in distinguishing single-amino-acid changes in peptide ligands that, otherwise, would likely be difficult to detect using GPCRs alone. Proteases could irreversibly cleave peptides differing only in a single-amino-acid and prevent binding of such peptides to cognate GPCRs. Hence, we have a dual biosensor system where the first strain without the protease would confirm the presence of both single-amino-acid variants that would both activate the GPCR, and the second strain with the protease would distinguish between the variants by cleaving only one variant and prevent activation of GPCR in that strain.

We established a proof of concept of such a living biosensing system by implementing and testing natural fungal GPCRs, proteases, and peptides engineered laboratory yeast strain. We previously developed a peptide/GPCR communication language where we determined the functionality of 30 fungal GPCRs, which were heterologously expressed in laboratory yeast and activated with unique linear pheromone peptides [5]. To introduce proteases into the biosensor, we looked to implement pheromone proteases that are native to these fungal species. We identified proteases from 5 fungal species that we could implement in our biosensor along with their corresponding GPCRs and peptides: *S. cerevisiae*, *Candida albicans*, *Schizosaccharomyces pombe*, *Schizosaccharomyces octosporus*, and *Schizosaccharomyces japonicus* (Table). *S. cerevisiae* [6], *C. albicans* [7], and *S. pombe* [8] pheromone proteases were reported and characterized previously as essential components in fungal mating by cleaving their corresponding pheromones and creating pheromone gradient that guides mating partners toward each other. *S. octosporus* and *S. japonicus* putative proteases were selected because of high homology to *S. pombe* protease. In a laboratory setting, pheromone proteases shift the apparent EC_50_ (concentration of peptide required for half-maximal activation) toward higher pheromone peptide concentrations in standard GPCR dose–response activation assays, as greater peptide concentration is needed for GPCR activation to overcome protease cleavage threshold (Fig. 1B).

**Table.** Summary of functional fungal peptide/GPCR/protease pairs.

**Fungal species**	**Peptide**	**GPCR**	**Protease**
*S. cerevisiae*	ScPep	ScSte2	ScBar1
*C. albicans*	CaPep	CaSte2	CaBar1
*S. pombe*	SpPep	SpSte2	SpSxa2
*S. japonicus*	SjPep	SjSte2	SjSxa2
*S. octosporus*	SoPep	SoSte2	SoSxa2

The straightforward route to systematically determine single-amino-acid peptide variants with desired properties is alanine (Ala) scanning mutagenesis. Alanine scanning is a well-established and commonly used method to study protein–protein interactions where individual amino acid residues within the binding region are replaced with alanine to determine their contribution to the binding affinity. Alanine is used as a replacing amino acid since it eliminates the side chains, but it does not alter the main chain conformation [9]. In addition, alanine is the most abundant amino acid present in both buried and exposed positions, as well as in plethora of secondary structures [10]. Alanine scanning has also been used to study fungal pheromone peptide binding to fungal GPCRs and their cleavage by proteases [11,12]. Here, we applied alanine scanning to probe pheromone peptide variants that activate cognate GPCRs at similar extent as the natural peptides and to determine protease cleavage of those variants.

Here, we demonstrate the utility of a live yeast biosensor that coexpresses fungal proteases to both detect and distinguish peptides differing in a singl-amino-acid. We first established a genetic toolkit for straightforward mixing and matching of different fungal GPCRs, proteases, and readouts optimized engineered laboratory yeast strain. Then, we determined the expression level, functionality, and orthogonality of 5 selected fungal proteases using dose–response assays (Table). We then moved to systematically screen single-amino-acid effects of *S. cerevisiae* and *C. albicans* peptides on cognate GPCR activation and protease cleavage using alanine scanning. We aimed to find single-amino-acid-differing peptide pairs that both activate the GPCR at similar concentration range, but only one is cleaved by the protease (Fig. 1C). Ultimately, we were able to both detect and confirm specific point mutations in fungal pheromone peptides through a simple color change of yeast expressing lycopene as a visible readout (Fig. 1D). With these results, we established a proof-of-concept single-amino-acid peptide variant detection using a live yeast biosensor and a visible readout. The ability to readily detect single-amino-acid changes could have myriad applications including reporting variant viruses and other diseases caused by point mutations.

## Materials and Methods

### Strains and media

The *Escherichia coli* [New England Biolabs (NEB), C3040] was used for electrocompetent cell generation and was selected and grown in Luria broth (LB) medium and supplemented with ampicillin (100 μg/ml) or chloramphenicol (34 μg/ml). All *E. coli* used for cloning experiments were grown at 37 °C with shaking and aeration.

Yeast cells were cultured in yeast extract, peptone, and dextrose (YPD) medium at 30 °C and 200 rpm [13]. Transformation selections were performed in synthetic complete medium with glucose (SC/Glc) with uracil (20 mg/liter; Sigma-Aldrich), leucine (100 mg/liter; Sigma-Aldrich), or histidine (20 mg/liter; Sigma-Aldrich) depending on the auxotrophic selection. The cells were then grown overnight in liquid SC/Glc, shaking at 200 rpm in 30 °C, and then grown in solid SC/Glc static at 30 °C [13]. During the treatment, the yeast cells were cultured and grown in 200 μl of SC/Glc medium in a 96-well plate at 30 °C in a Glas-Col high-frequency shaker, shaking at 800 rpm. For plasmid transformations, yeast cells were transformed using lithium acetate protocol as described previously [13]. List of used yeast strains is available in Table S1.

### Materials

The *S. cerevisiae* α-factor (ScPep) was purchased from Zymo Research (Irvine, CA, USA), and other synthetic peptides were purchased from GenScript (Piscataway, NJ, USA) at ≥95% purity. Stock synthetic peptide solutions were prepared by resuspending peptide powder in sterile Mili-Q H_2_O. gBlocks and primers were purchased from Integrated DNA Technologies (Coralville, Iowa, USA), and plasmids were cloned and amplified in *E. coli* (C3040) (NEB). Reaction and assembly enzymes [polymerase chain reaction (PCR), Gibson Assembly, restriction digest, and Golden Gate Assembly (GGA)] were purchased from NEB (Ipswich, MA, USA). The 96-well microtiter plates used for yeast growth were purchased from Corning Inc.

### Construction of communication cassette plasmids and yeast genomic integration plasmids

List of all used plasmids is available in Table S2. List of expression modules and cassette sequences is available in Table S3.

The communication cassette plasmids are based on pYTK009 (*ori*, CmR selection marker) from MoClo Yeast Toolkit [14]. Cassette plasmids having individual expression cassettes with biosensor parts (constitutive GPCR, peptide-inducible readout, or constitutive protease) were cloned using Gibson Assembly and further on matched using GGA. Yeast genomic integration plasmid is an acceptor plasmid (*ori*, AmpR selection marker) into which biosensor component cassette plasmids are being assembled via GGA. The integration plasmid contains appropriate 500-base-pair homology sequences for genomic integration (ARS208a, HO or LEU2 locus), integration selection marker (LEU2, HIS3, or URA3), red fluorescent protein expression cassette that gets replaced with correctly assembled biosensor parts, and unique Not I restriction sites for repair fragment linearization.

### GGA of communication cassettes and CRISPR-Cas9 genomic integration

A Golden Gate reaction mixture with appropriate communication cassettes and integration plasmid were prepared and incubated according to the manufacturer’s protocol. The mixture is then diluted 1:3 with water, and electrocompetent *E. coli* is transformed via electroporation and plated on LB/Amp plates. Correctly assembled plasmids are screened and verified using colony PCR and Sanger sequencing. Correctly transformed colony was grown in LB/Amp overnight and stocked with 20% glycerol at −80 °C.

In a typical genomic integration of assembled integration plasmids using CRISPR-Cas9, the plasmids were digested with Not I restriction enzyme and directly used along with Cas9/guide RNA (gRNA) plasmid (URA3 or NAT selection marker) following lithium acetate yeast transformation method. Transformed colonies are grown on selective medium for the selection marker integrated along with the remaining biosensor components and for the Cas9/gRNA plasmid. Correctly integrated colonies are screened phenotypically when possible, by activating integrated components with appropriate synthetic peptide and following appropriate fluorescence/color development, and sequence is verified using PCR amplification and Sanger sequencing from purified genomic DNA of the screened colony. Transformed colony was cured from Cas9/gRNA plasmid by growing in nonselective medium and replica plating. Single cured colony was grown in YPD overnight and stocked with 15% glycerol at −80 °C. CRISPR-Cas9 gRNA sequences are available in Table S4.

### Biosensor activation assay and dose–response assay using fluorescent readout

Glycerol stocked strains were streaked out on YPD plate and incubated for 2 days. Three colonies were picked, replated on YPD plate, incubated for 1 day, and then stored at 4 °C. For activation assays, a small scoop of yeast patch from the 3 colonies was resuspended in ~4 ml of YPD medium and incubated overnight. Overnight cells were seeded at an optical density at 600 nm (OD_600_) of 0.15 (as measured in 96-well plate at 200-μl volume) in SC/Glc medium after washing twice with sterile Mili-Q H_2_O. GPCR activity and response to increasing concentrations of synthetic peptide ligands were measured in strains carrying appropriate genome-integrated components in 96-well microtiter plates in 200-μl total volume and cultured at 30 °C and 800 rpm, unless otherwise stated. All measurements were performed in biological triplicate. Culture turbidity (OD_600_) and fluorescence for ymTurquoise2 (excitation, 439 nm; emission, 475 nm; gain, 75) were measured after 0 and 8 h using an Infinite 200 Pro plate reader (Tecan) [4]. Dose–response curve was measured at 11 different concentrations of the appropriate synthetic peptide ligand with a constant dilution factor and a no-peptide, H_2_O control. The absorbance at 600 nm normalized fluorescence values and log_10_-transformed peptide concentrations were fit to a 4-parameter nonlinear regression model using Prism (GraphPad) to extract GPCR-specific values for basal activation, maximal activation, EC_50_, and the Hill coefficient.

### Biosensor activation assay and dose–response assay using lycopene readout strains

Induction of lycopene was assayed in transparent 96-well microtiter plates cultured at 30 °C and 800 rpm. Cells were measured in SC/Glc medium for an OD_600_ of 2 for seeding. All measurements were performed in biological triplicate. Relative lycopene content was calculated by spectroscopy as described previously [4]. Optical densities were measured with an Infinite M200 plate reader (Tecan). Lycopene values were normalized by the culture OD_600_ to give a measure of lycopene per cell.

### Quantification and statistical analysis

Statistical tests of all experiments were performed using GraphPad Prism version 7 and are detailed within the legend of each figure. In all figures, the data points represent means ± SD of biological triplicates. Curves fitted to all dose–response data were fitted in Prism 7 using the nonlinear regression: variable slope (4-parameter) curve fitting.

## Results

### Building modular genetic toolkit for facile assembly of biosensing components into engineered yeast

To identify proteases that could distinguish wild-type and variant peptides, we first needed a toolbox of functional fungal proteases that cleave their native mating peptides. We first identified characterized fungal pheromone proteases from the literature. *S. cerevisiae*’s Bar1 aspartyl protease (ScBar1) [6], *C. albicans*’ Bar1 aspartyl protease (CaBar1) [7], and *S. pombe*’s Sxa2 serine carboxypeptidase (SpSxa2) [8] have all been previously reported, so, first, we cloned these proteases and synthesized their corresponding pheromone peptides. Through inspection of sequence homology to these proteases, we further identified 2 additional putative pheromone proteases: *S. japonicus*’ serine carboxypeptidase Sxa2 (SjSxa2; 43% identity to SpSxa2) and *S. octosporus*’ serine carboxypeptidase Sxa2 (SoSxa2; 60% identity to SpSxa2). The complementary pheromone GPCRs (ScSte2, CaSte2, SpSte2, SjSte2, and SoSte2, respectively) were all cloned and reported previously and the orthogonal pheromone peptides (ScPep, CaPep, SpPep, SjPep, and SoPep, respectively) were all reported by our laboratory and made by standard solid-phase peptide synthesis [5]. The fungal peptide/GPCR/protease nomenclature is summarized in Table. The amino acid sequences of these 5 proteases, GPCRs, and peptides are given in Tables S5, S6, and S8, respectively.

The next step was to engineer yeast biosensor strains having all the necessary components to test the functionality of the proteases—the protease, the GPCR, and the fluorescent reporter. We started by genetically modifying strain yWS890 engineered by Shaw *et al.* [13], which has a remodeled and optimized pheromone signaling pathway. The yWS890 strain has a native *S. cerevisiae* pheromone GPCR and a pheromone-inducible expression of superfolder green fluorescent protein (sfGFP). To make a parent yWS890 strain where we can modularly combine the GPCR, protease, and a readout of our choice, we knocked out the Ste2, sfGFP, LEU2, and URA3 along with their corresponding promoters and terminators. LEU2 and URA3 selection markers were deleted, since their auxotrophy is needed for the CRISPR-Cas9 genomic integration selections (see Materials and Methods). The engineered parent yeast strain (yTC370) has a remodeled and optimized pheromone signaling pathway and is lacking only the 3 expression components (GPCR, inducible readout, and protease) to complete the functional peptide-inducible GPCR response system. Then, we used the parent strain to engineer yeast that express all relevant biosensing components (GPCR, fluorescent or lycopene readout, and protease).

To simply mix-and-match desired combinations of the protease and the GPCR, we cloned codon-optimized open reading frames (ORFs) of all 5 proteases and GPCRs along with appropriate promoters and terminators into standard GGA (see Materials and Methods) cassette plasmids using Gibson assembly (Tables S3 and S7). We selected strong constitutive promoters for GPCRs and proteases and orthogonally inducible LexO(6x)-pLEU2m promoter for ymTurquoise2 fluorescent protein readout expression. For all ORFs, standard characterized yeast terminators were selected [14]. The cassette plasmids are a modification of the MoClo Yeast Toolkit plasmids [14] where each cassette plasmid contains a full transcriptional unit that can be modularly assembled into a multigene plasmid using GGA. The cassette plasmids were constructed to assemble a complete activation pathway (constitutive GPCR expression, inducible readout expression, and constitutive protease expression) into a genomic integration plasmid that can be transformed into parent yeast strain yTC370. Sequences of promoters, terminators, ORFs, and GGA overlaps are available in Table S3.

### Fungal peptide proteases are selective for their cognate fungal pheromones

**Fig. 2.**
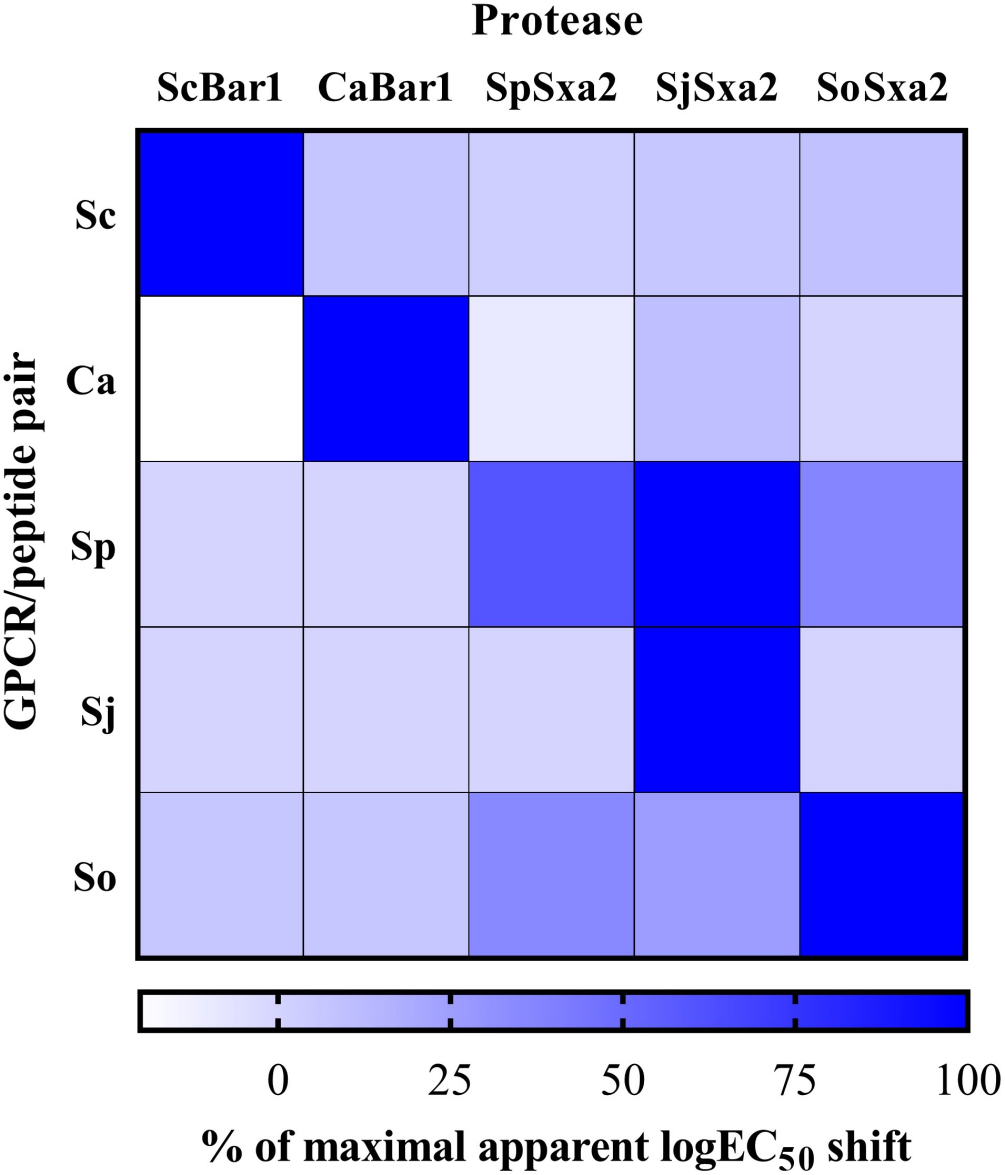
Proteases are orthogonal across noncognate GPCR/peptide activation pairs. The maximal apparent shift in logEC_50_ with each protease (not necessarily the cognate protease) was set to 100% shift. All experiments were run in biological triplicate.

To validate the effect of peptide proteases on their complementary and noncomplementary peptides (Table S8), from the parent strain yTC370, we engineered 30 strains (Table S1). These strains all have the same peptide-inducible fluorescent readout (ymTurquoise2), and they have all possible combinations of complementary and noncomplementary GPCRs and proteases, including strains that only have the GPCR and do not have any protease. We then generated dose–response curves with GPCR-complementary peptides from which we can detect the apparent shift in EC_50_ toward higher peptide concentration in an instance when the protease effectively cleaves the peptide and prevents it from activating the GPCR within a particular peptide concentration range (Fig. 2 and S1).

For *S. cerevisiae*, *C. albicans*, and *S. japonicus* peptide/GPCR pairs, only their complementary proteases (ScBar1, CaBar1, and SjSxa2, respectively) shifted the apparent EC_50_ by over one order of magnitude. Other noncomplementary protease combinations did not substantially affect apparent EC_50_. For *S. pombe* and *S. octosporus* pairs, their cognate proteases (SpSxa2 and SoSxa2, respectively) strongly shifted the apparent EC_50_. SpSxa2 practically completely attenuated the response with SpPep within measured concentration range, and SoSxa2 shifted the apparent EC_50_ by one and a half orders of magnitude toward higher SoPep concentration. However, in *S. pombe* and *S. octosporus* pairs, 2 noncomplementary proteases (SjSxa2 and SoSxa2 and SjSxa2 and SpSxa2, respectively) also shifted the apparent EC_50_ between half and one order of magnitude, while ScBar1 and CaBar1 did not cleave SpPep and SoPep. For *S. pombe* and *S. japonicus* peptide/GPCR pairs, the complementary proteases (SpSxa2 and SjSxa2, respectively) shifted the apparent EC_50_ out of the measurable peptide concentration range (>100 μM) (S1). Our results confirm the specificity of fungal pheromone proteases to their native pheromone peptides as discussed in previous studies [12]. The cross-cleavage of noncognate peptides only remarkably activated receptors with similar cognate peptides (S6), evident by the cross-cleavage results of SoSxa2/SjSxa2 for SpPep and SjSxa2/SpSxa2 for SoPep.

### Protease’s promoter strength increases the extent of apparent EC_50_ shift of cognate peptides

Next, to validate whether we can control the extent of peptide cleavage by their cognate proteases by modulating the concentration of expressed protease, we constitutively expressed proteases under promoters of different expression levels. For the purpose of determining the protease’s specificity in previous section (S1), all proteases were expressed under pCCW12, which is one of the strongest characterized promoters in yeast [15]. Here, we introduced 2 additional promoters to convey medium and low expression levels of the proteases. We selected pRPL18B for medium expression and pRAD27 for low expression level [15]. We cloned these promoters upstream from protease ORFs in Golden Gate cassette plasmids, assembled the protease cassettes with their cognate GPCRs and fluorescent readout, and integrated into parent yTC370 strain (see Materials and Methods and Table S1). Then, we compared dose–response curves of strains responding to their cognate peptides without the protease or with protease at 3 different expression levels (low, medium, and high) (S2). Overall, for all tested proteases, stronger expression level led to greater apparent EC_50_ shift. For ScPep and CaPep, medium and high expression levels of the cognate proteases ScBar1 and CaBar1, respectively, did not show remarkable difference in the extent of EC_50_, and for both, low protease expression level showed EC_50_ shift between the no-protease response and medium/high protease response. For SpPep, low protease did not show notable apparent EC_50_ shift, medium protease shifted EC_50_ almost 2 orders of magnitude, and high protease expression practically completely attenuated the response. For SoPep, low and medium protease expression levels did not notably shift the apparent EC_50_ compared to strain with no protease; however, high protease shifted the apparent EC_50_ around one order of magnitude. Finally, for SjPep, low protease expression level did not affect EC_50_, while medium and high protease expressions gradually shifted the apparent EC_50_ more than 2 orders of magnitude out of the measurable peptide concentration range (>100 μM).

Finally, we tested whether integration of additional copies of ScBar1 and CaBar1 would lead to a greater shift in the apparent EC_50_, as this would improve the working peptide concentration range where the activation difference between +protease and −protease strain could be distinguished (Fig. 1C). In the *S. cerevisiae* and *C. albicans* peptide/GPCR pair strains with their corresponding cognate proteases (yTC398 and yTC399, respectively), an additional copy of the strong constitutive protease expression cassette was integrated into the HO locus (yBB01 and yBB02, respectively) (see Materials and Methods and Table S1). Although the dose–response curves in S3 show that the additional protease copy marginally shifts the apparent EC_50_ compared to a single copy, EC_50_ was shifted nevertheless, so further experiments were done with these strains.

### ScPep alanine scanning shows high tolerance of point mutations in both GPCR activation and protease cleavage

**Fig. 3.**
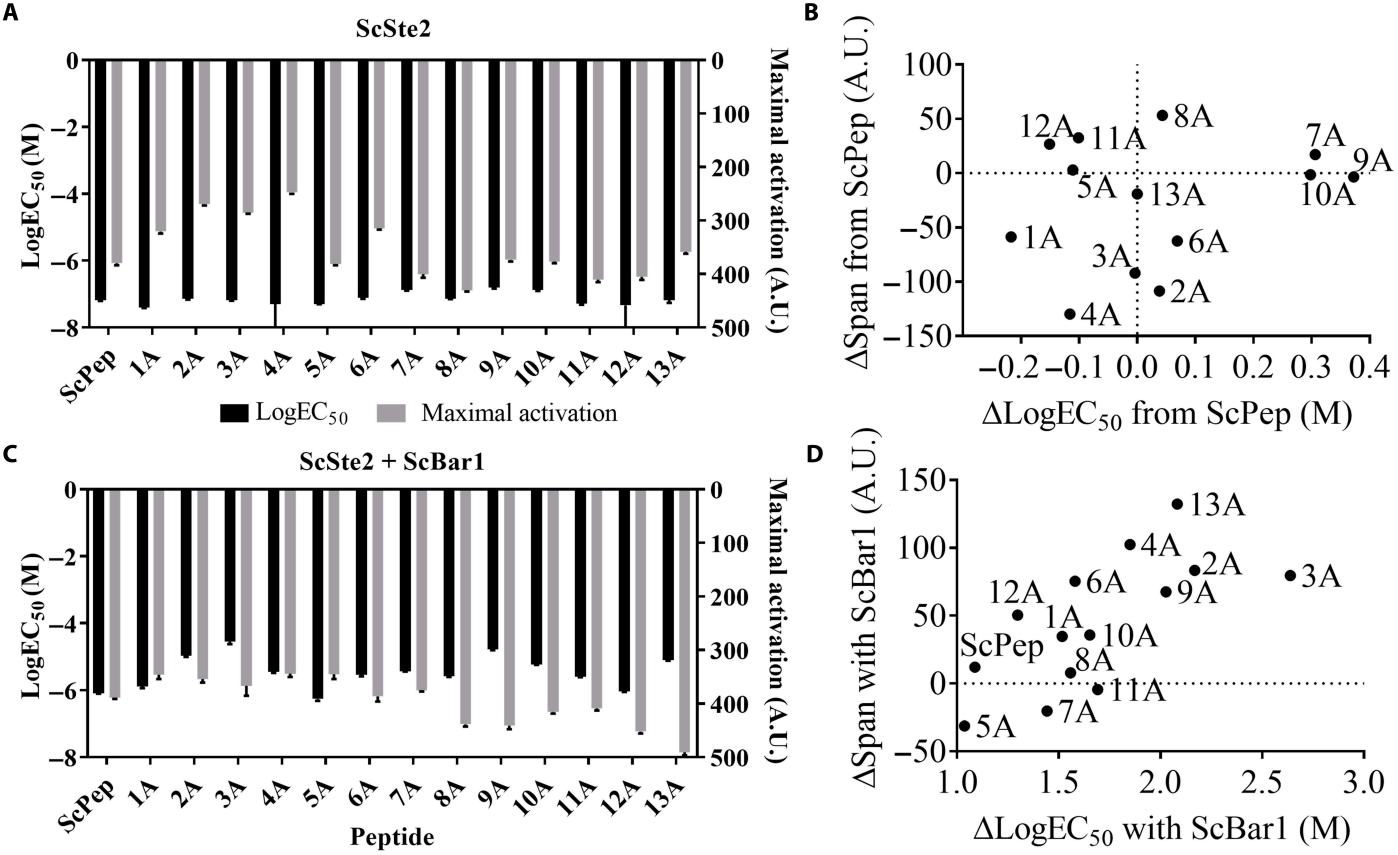
ScPep alanine scanning show high tolerance of point mutations in both GPCR activation and protease cleavage. (A) ScPep alanine scanning logEC_50_ and maximal activation values without secreted cognate protease ScBar1. (B) Change in activation span (variant peptide Span − ScPep Span) and logEC_50_ (variant peptide logEC_50_ − ScPep logEC_50_) of each peptide variant compared to ScPep. (C) ScPep alanine scanning logEC_50_ and maximal activation values with secreted cognate protease ScBar1. (D) Change in activation span (peptide Span with ScBar1 − peptide Span without ScBar1) and apparent logEC_50_ (peptide logEC_50_ with ScBar1 − peptide logEC_50_ without ScBar1) of each peptide due to ScBar1 expression. All experiments were run in biological triplicate, and error bars represent the standard deviation.

A previous report of an alanine scanning of the CaPep suggested that peptide/GPCR/protease systems may be able to distinguish single-amino-acid changes in the peptide epitope [16]. To test this hypothesis, we systematically measured the effect of single-site alanine substitutions in ScPep and CaPep on the complementary GPCR activation and protease cleavage (Figs. 2 and 3) [9]. We validated dose–response activation of yTC391 (ScSte2) and yBB01 (ScSte2 + ScBar1) with ScPep and single alanine-substituted variants and yTC384 (CaSte2) and yBB02 (CaSte2 + CaBar1) with CaPep and alanine-substituted variants by measuring ymTurquoise2 fluorescence intensity. Changes in EC_50_ and activation span (maximal fluorescence activation − basal fluorescence activation) due to difference in variant peptide sequences from the wild-type peptides are depicted as ΔlogEC_50_ from wild-type Pep (variant peptide logEC_50_ − wild-type peptide logEC_50_) and ΔSpan from wild-type Pep (variant peptide Span – wild-type peptide Span), respectively. Apparent shift in EC_50_ and change in activation span in both wild-type and variant peptides due to protease expression are depicted as ΔlogEC_50_ with protease (peptide logEC_50_ with protease − peptide logEC_50_ without protease) and ΔSpan with protease (peptide Span with protease − peptide Span without protease), respectively. Alanine-scanned peptide sequences and measured Hill function parameters that are discussed below are in Tables S9 and S10.

For the Sc peptide/GPCR pair (Fig. 3), the original ScPep and single-site alanine variants activated cognate GPCR with EC_50_ ranging between 40 and 160 nM. The maximum fluorescent intensity of the variants was no more than 30% value of the original ScPep. When we coexpressed ScBar1 protease, we observed different GPCR responses compared to the initial system. Original ScPep and all variant peptides have shifted apparent EC_50_ to higher peptide concentrations compared to system without ScBar1. The shift ranged between 1 and 2.5 orders of magnitude toward higher peptide concentration. Interestingly, the maximal activation increased for most of the variants. Introduction of ScBar1 also affected the Hill slope values. In sensors, the Hill slope describes their ligand concentration operational range with lower Hill slope values meaning higher operational concentration range and higher Hill slope values meaning narrower operational concentration range where the sensor responds in a switch-like manner [13]. ScBar1 greatly decreased Hill slope values for ScPep and all its variants where dose–response curves showed more gradual response over wider peptide concentration range.

We previously showed that many fungal GPCRs expressed in yeast are highly orthogonal to their cognate peptides [5]. However, in agreement with previous studies, the GPCRs were still activated after the introduction of most point mutations. Houen et al. [17] showed that substitutions in ScPep in positions 1, 5, and 7 retained its pheromone activity in growth inhibition assay. Siegel et al. [18] showed that substitutions in ScPep in positions 12 and 12 + 13 showed similarly strong growth inhibition as the native ScPep, while substitution in position 1 led to lower inhibition. In the most extensive study on variant ScPep activity, Abel et al. [11] performed a systematic alanine scanning where all residues in ScPep were replaced and showed that all l-enantiomeric variants were able to illicit growth inhibition in ScPep-responsive yeast strain [11]. Last, Velazhahan et al. [19] characterized ScPep bound to ScSte2 using cryo-electron-microscopy and determined that ScPep residues 2, 4, and 13 have the greatest number of interactions in the binding pocket of ScSte2. However, replacing those residues with alanine has not greatly changed the activation in our results (Fig. 3).

### CaPep alanine scanning shows single-amino-acid-dependent activation by GPCR and cleavage by protease

**Fig. 4.**
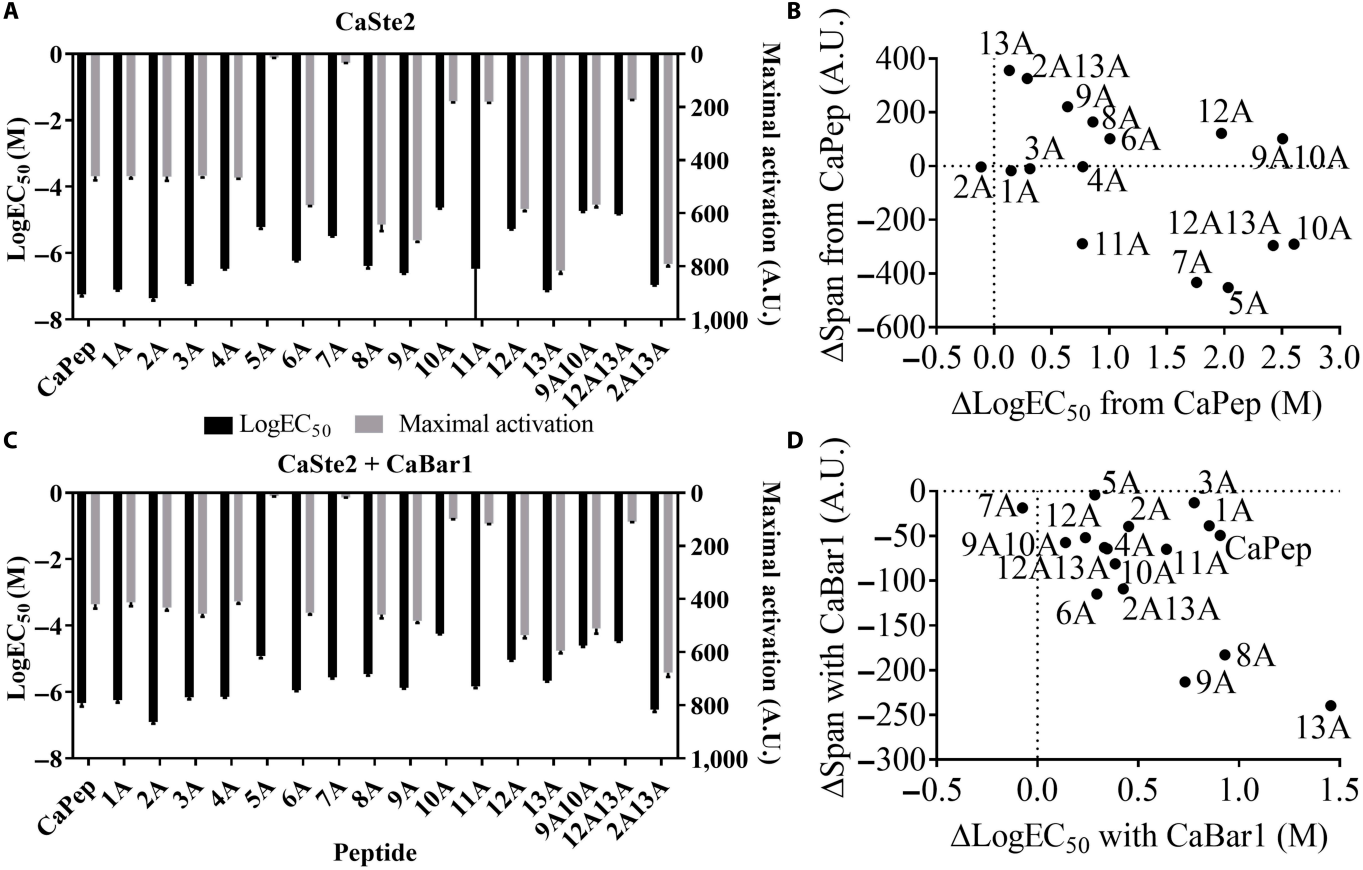
CaPep alanine scanning show single-amino-acid differences in GPCR activation and protease cleavage. (A) CaPep alanine scanning logEC_50_ and maximal activation values without secreted cognate protease CaBar1. (B) Change in activation span (variant peptide Span − CaPep Span) and logEC_50_ (variant peptide logEC_50_ − CaPep logEC_50_) of each peptide variant compared to CaPep. (C) CaPep alanine scanning logEC_50_ and maximal activation values with secreted cognate protease CaBar1. (D) Change in activation span (peptide Span with CaBar1 − peptide Span without CaBar1) and apparent logEC_50_ (peptide logEC_50_ with CaBar1 − peptide logEC_50_ without CaBar1) of each peptide due to CaBar1 expression. All experiments were run in biological triplicate, and error bars represent the standard deviation.

Alanine scanning of Ca peptide/GPCR pair showed greater disparity in single-amino-acid effects in GPCR activation compared to Sc peptide/GPCR pair (Fig. 4). In a system without the CaBar1 protease, variants CaPep5A and CaPep7A are practically nonresponsive, while CaPep10A, CaPep12A, CaPep9A10A, and CaPep12A13A have more than 2 orders of magnitude higher EC_50_ compared to the native CaPep. CaPep11A has the maximal activation reduced by 50% compared to the native CaPep. GPCR activation in the presence of CaBar1 for other functional variants shows apparent shifts in EC_50_ between zero to one order of magnitude on top of already shifted EC_50_ in no-protease system due to peptide sequence differences. Like in the Sc alanine scanning, introduction of CaBar1 led to decrease in Hill slope values for the CaPep and the majority of its variants. In both Sc and Ca alanine scanning, bottom fluorescence values fitted to Hill function have small differences for each peptide ranging from −4.4 to 17 (Tables S9 and S10). These differences are solely due to fitting variations, as the basal fluorescence value is determined at peptide concentration zero and this fluorescence value is independent of the peptide sequences. The basal fluorescence value of 4 yeast strains tested in Sc and Ca alanine scanning (yTC391, yTC384, yBB01, and yBB02) is 5 ± 1 arbitrary units. In addition, because of large slope of the curve and too few concentration points around the curve of inflection point, logEC_50_ standard error for some peptides is extremely high (ScPep4A, ScPep12A, and CaPep11A), but the average EC_50_ values were confident enough for the screening purpose of the alanine scanning.

A previous study by Alby and Bennett [16] demonstrated diminished peptide induction of GFP expression in a CaPep dialanine scanning. We confirmed a similar result with 2 disubstituted peptides: CaPep9A10A and CaPep12A13A. As expected, the double alanine substitutions produced lower activity than a single alanine substitution. However, we also found that neighboring amino acids within CaPep can yield great in activation. Both N- and C-terminal mutations were less prone to CaBar1 cleavage. In addition, a CaPep mutation in position 2 rendered the noncleavable peptide and retained that property in the CaPep2A13A variant of the cleavable CaPep13A (Fig. 4). On the basis of these results, we selected native CaPep and CaPep variants CaPep2A, CaPep13A, and CaPep2A13A as suitable candidates for further validation of a biosensor for the distinction of single-amino-acid difference within peptides (CaPep versus CaPep2A and CaPep13A versus CaPep2A13A) in visible readout strains.

### Engineered biosensor system expresses pigment lycopene as a visible readout

To demonstrate that variant detection and distinction can be achieved in our live yeast biosensor with readout visible to the naked eye, we engineered biosensing strains that have carotenoid pigment lycopene as a readout, instead of a fluorescent protein. First, on the basis of our previously reported yeast biosensor [4], we engineered a parent strain yTC412 analogous to yTC370 that has integrated all relevant genes in lycopene biosynthetic pathway. This strain is only missing the GPCR and protease component that are assembled using GGA and integrated using acceptor integration plasmid. Lycopene is a carotenoid pigment naturally produced by bacteria and plants. Lycopene can be biosynthesized in yeast from native precursor farnesyl pyrophosphate by introducing only 3 additional genes: geranylgeranyl diphosphate synthase (*CrtE*), phytoene synthase (*CrtB*), and lycopene synthase (*CrtI*).

To engineer lycopene parent strain yTC412, we integrated the first 2 biosynthetic genes *CrtE* and *CrtB* under strong constitutive promoters and 2 copies of the last gene lycopene synthase (*CrtI*) under control of peptide-inducible promoter into yTC370. In addition, we integrated a copy of the endogenous flavin adenine dinucleotide synthase (*FAD1*) to achieve faster color development as demonstrated in our previous biosensor (see Materials and Methods and Table S3) [4]. This strain constitutes a complete peptide-inducible lycopene biosynthetic pathway, and the final component needed to achieve peptide biosensing is the receptor and the protease. We integrated CaSte2 without CaBar1 or with 2 copies of CaBar1 (yTC646 and yTC682, respectively) into this parent biosensor strain.

### Preincubation of lycopene readout strains shows apparent EC_50_ shift of more than one order of magnitude in protease-sensitive peptides

Next, we tested lycopene strain activation with selected peptides from the CaPep alanine scan (CaPep, CaPep2A, CaPep13A, and CaPep2A13A). The strain dose–response activation was measured by absorbance and visual inspection of the wells after incubation with each of the selected peptides at different concentrations. In addition, we validated whether preincubation of the strains before adding the peptides would more effectively shift the apparent EC_50_ in the strain expressing the protease to increase the working peptide concentration range. Dose–response assay with a preincubation step involved resuspending appropriate sensing yeast in medium in the 96-well plate and incubating it with shaking for 1 h. Then, peptides at appropriate concentrations were added to the wells and the incubation continued. We hypothesized that with preincubation, the secreted protease concentration would build up in the medium and have greater catalytic activity once the peptide is added, thus shifting the apparent EC_50_ more strongly.

**Fig. 5.**
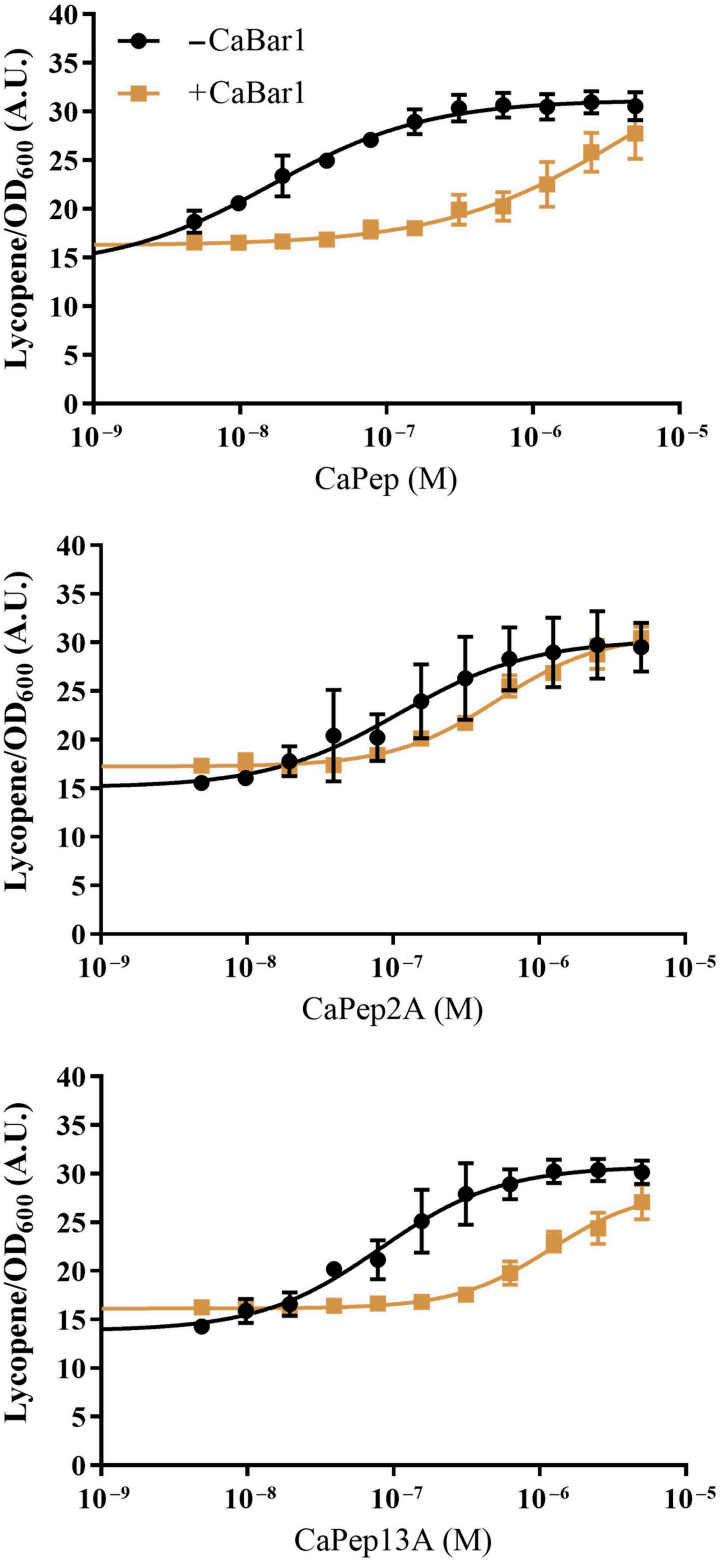
CaBar1 protease in biosensor strain with lycopene readout greatly shifts the apparent EC_50_ of CaPep and CaPep13A, but not CaPep2A in liquid culture. Peptides were added to cells after 1-h preincubation, and then the measurements were taken after 8-h incubation. All experiments were run in biological triplicate, and error bars represent the standard deviation.

First, we validated lycopene strain activation and color development in liquid culture with 4 selected peptides (CaPep, CaPep2A, CaPep13A, and CaPep2A13A). CaPep2A and CaPep2A13A (noncleavable peptides) had similar response in both −CaBar1 and +CaBar1 strains with EC_50_ between 120 and 250 nM and similar maximal activation intensity (S4). However, CaPep and CaPep13A (cleavable peptides) had apparent EC_50_ shifted by at least 3-fold in +CaBar1 strain (from 160 to 450 nM and from 66 to 330 nM, respectively). To make the apparent EC_50_ shift more prominent and to expand working concentration range where we can distinguish a single-amino-acid difference, we preincubated biosensor strains before adding the peptides. This setup led to ~260-fold and 15-fold shifted apparent EC_50_ in +CaBar1 lycopene strain toward higher concentration when activated with CaPep and CaPep13A, respectively (17 to ~4,400 nM and 80 to 1,200 nM, respectively). The noncleavable variant CaPep2A showed a moderate shift in apparent EC_50_ from 110 to 520 nM (Fig. 5 and S5). The preincubation greatly improved the working concentration range where the cleavable and noncleavable peptide variants could be distinguished.

### Living yeast biosensor plus protease can visibly detect and distinguish wild-type and variant peptide in yeast pellets

**Fig. 6.**
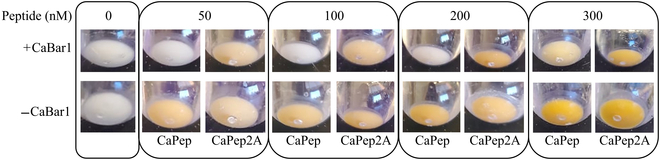
Yeast pellets detect and distinguish between 2 peptides differing in single-amino-acid in their sequence. Cultures (2 ml) of Ca-responsive biosensor strains with lycopene readout and with (+CaBar1) or without (−CaBar1) expressed protease were incubated for 1 h before adding CaPep or CaPep2A at appropriate concentrations. Photos of the representative pelleted cultures were taken after 4-h incubation. All experiments were run in biological triplicate.

Encouraged by these results, we then moved our efforts to visually inspect the color change in concentrated yeast pellets with CaPep and CaPep2A, since these peptides showed the strongest activation difference with the protease strain tested in 96-well cultures from the previous section (Fig. 5). We incubated Ca-responsive biosensing strains with or without the CaBar1 protease (yTC682 and yTC646, respectively) in transparent culture tubes for 1 h and then added either CaPep or CaPep2A in nanomolar concentration range. The cultures were incubated for another 4 h and then centrifuged to observe the color change in the yeast pellets. Using this assay, we observed a robust orange color development in −CaBar1 strain with both tested peptides, and we observed color in +CaBar1 strain only with CaPep in the peptide concentration range of 50 to 300 nM, thus distinguishing the single-amino-acid difference between CaPep and CaPep2A (Fig. 6).

## Discussion

Our results established a living yeast biosensor with reintroduced fungal mating protease that enabled distinction of single-amino-acid peptide variants with apparent EC_50_ difference of at least one order of magnitude between selected peptide variants without additional GPCR or protease design or optimization. A shift of more than two orders of magnitude was observed for CaPep, and a shift of more than one order of magnitude was observed for CaPep13A in lycopene biosensor strain with the protease compared to the strain without the protease. Meanwhile, CaPep2A showed less than 5-fold shift in the same biosensor system that enabled CaPep2A distinction from the CaPep in a nanomolar concentration range in proof-of-concept yeast pellets assay (Fig. 6).

As we developed our living yeast biosensor technology, we also acquired a fundamental understanding of fungal mating components and how they can be used as synthetic biology tools. On top of already established peptide/GPCR communication language, where fungal peptides can specifically activate their cognate GPCRs [5], fungal proteases can be implemented in an analogous way as specific negative regulators of their cognate fungal peptides and serve as GPCR activation modulators. We characterized 5 fungal proteases and their peptide cross-cleavage; however, the protease toolkit can be further expanded through genome mining. In such a way, we identified 2 putative carboxypeptidases from *S. octosporus* and *S. japonicus* that have not been characterized previously as cognate pheromone proteases and demonstrated their functional expression in laboratory yeast and highly specific cleavage of cognate pheromone peptides (S1).

Further on, we used alanine scanning to screen for fungal pheromone variants that could be distinguished using our biosensor system. As a proof of concept of our technology, we retroactively screened for the peptide variants that can be distinguished with well-characterized fungal GPCRs and proteases, while for future applications of our system, receptors and proteases would be screened and engineered toward detection and distinction of peptide variants of interest. Furthermore, potentially any peptide or protein variant could be distinguished using our system through rational design of mutation-permissive binding events and mutation-specific irreversible cleavage events. Already established receptors and antibodies, as well as newly engineered ones, can be used as a general platform for detection of ligands and epitopes, and proteases from existing protease databases [20] or rationally designed ones using established engineering methods [21,22] can be implemented for mutation distinction.

The binding of GPCRs and peptide ligands is based on complex molecular recognition; however, oftentimes, the receptor can tolerate point mutations in the ligand that was also evident in our results (Figs. 3 and 4). Biosensors and diagnostics that rely on binding events would not readily be able to distinguish single-amino-acid changes within the contact residues, as previous studies showed that point mutations are highly tolerated in binding of both ligands to receptors and antigens to antibodies. For example, binding of human growth hormone (hGH) to the extracellular domain of its first bound receptor involves around 30 amino acid residues; however, only a few of those residues account for the majority of the binding free energy [23]. Similarly, binding of hGH to anti-hGH monoclonal antibodies is primarily determined by only 5 residues, and substitutions of any other neighboring residues do not remarkably change binding affinity [24,25]. By introducing an irreversible cleavage by a single residue-specific protease, we can improve on the small binding affinity changes in most of the contact residues and achieve more diverse single-residue distinction in binding-based biosensors.

Nucleic-acid-based diagnostics have already employed endonucleases to distinguish single-nucleotide polymorphism in PCR-based diagnostic [26]. Here, the pheromone proteases, rather than nucleases, are used to distinguish single-amino-acid variants in GPCR-based biosensor. Our proof-of-concept application toward peptide targets can be more broadly applied to other antibody-based diagnostics to provide variant detection. We used alanine scanning to validate our system, since it is a standard method for systematic point mutation validation in peptide ligands and their binding to receptors [9]. However, our system can be repurposed for other amino acids or multiple point mutations potentially present in the variant. Here, we used natural fungal GPCRs and proteases for simplicity, but in a diagnostic device, both fungal and nonfungal GPCRs and proteases can be engineered toward new epitopes and for enhanced activity (e.g., expanded and clinically relevant concentration ranges or greater signal-to-noise ratio). Currently available tools and technologies enable extensive engineering of the GPCR/protease system toward detection of new epitopes and identification of their variants [27,28]. In fact, enzymes have been most extensively retooled using directed evolution [21,22,29]. For instance, multiple mutations could be detected simultaneously by engineering a system with 2 or more proteases with different mutation specificities. Moreover, our variant detection principle can be expanded to other engineered functionalities in living cells, such as variant sensing and responding by secretion of appropriate therapeutics. Finally, our prototype dipstick assay envisioned in Fig. 1D and developed in conjunction with our initial living biosensor could be used with complex biological matrices, as we demonstrated previously that peptide epitopes can be detected from soil, urine, serum, and blood [4].

Last, single-amino-acid variant distinction could be potentially used in point of care. With the progressing coronavirus disease 2019 pandemic, the majority of at-home severe acute respiratory syndrome coronavirus 2 diagnostics relied on detection of conserved nucleocapsid epitope to make such diagnostics continuously applicable with rapidly emerging viral variants [30]. However, with differences in severity and transmissibility of certain pathogenic variant diseases, distinction of variants can inform vaccination and therapeutic decision-making, as seen with new severe acute respiratory syndrome coronavirus 2 variants [31] and the porcine epidemic diarrhea virus [32]. Living biosensors offer distinct advantages of rapid scalability and ease of use compared to conventional diagnostics, allowing for swiftly deployable surveillance of emerging pathogens on a global scale [33].

## Conclusion

We expanded on our living yeast biosensor to include fungal protease for a single-amino-acid mutation distinction within pheromone peptide sequences. We characterized 5 fungal proteases with their corresponding GPCRs and peptides, and we used alanine scanning to systematically probe peptide sequence dependence on GPCR activation and protease cleavage of *S. cerevisiae* and *C. albicans* components. The *C. albicans* GPCR was activated to a similar extent by its native CaPep and 3 of its variants CaPep2A, CaPep13A, and CaPep2A13A, while its protease shifted the apparent EC_50_ of only CaPep and CaPep13A by more than one order of magnitude in a yeast biosensor system. This allowed us to establish a proof-of-concept variant detection system where a single-amino-acid difference in a fungal peptide was distinguished in nanomolar range using engineered living cells with visible color change as a readout. Despite advancement and momentum in the field of biosensor technology, living diagnostic biosensors are not yet available for detection of the variants and point mutations. Our prototype can be expanded to detect novel antigenic variants through rational design of both receptors and proteases.

## Data Availability

Data are freely available upon reasonable request. Plasmids and strains are available upon request under material transfer agreement.
