# Supplementary material for: Peptide Variant Detection by a Living Yeast Biosensor via an Epitope-Selective Protease
Source: Biodes Res. 2023 Mar 15;5:0003. doi: 10.34133/bdr.0003 (PMC10084949; doi:10.34133/bdr.0003)
Supplement: Supplementary 1 — Figs. S1 to S6 Tables S1 to S10 [file bdr.0003.f1.docx]

Front Matter

**Title**

Peptide Variant Detection by a Living Yeast Biosensor via an Epitope-Selective Protease

Short: Biosensor for Peptide Variant Detection

**Authors**

T. Crnković^1^, B. J. Bokor^2^, M. E. Lockwood^3^, V. W. Cornish^1,4^*

Affiliations

^1^Department of Chemistry, Columbia University, United States

^2^Department of Biological Sciences, Columbia University, United States

^3^School of General Studies, Columbia University, United States

^4^Department of Systems Biology, Columbia University, United States

*Corresponding author. Email: vwc114@columbia.edu

SUPPLEMENTARY MATERIALS


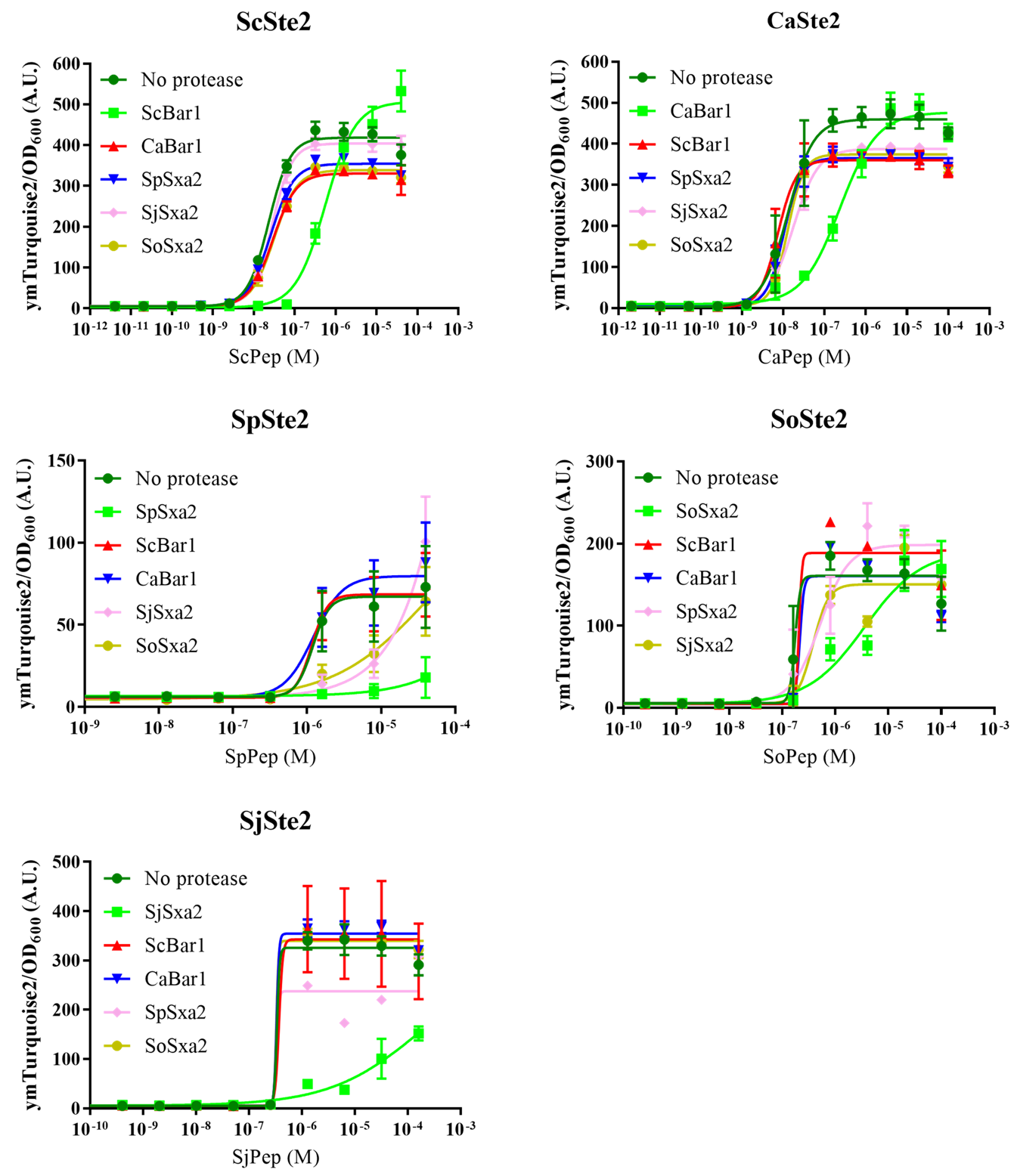


**Figure S1. Fungal peptide proteases are highly specific for their cognate peptides.** Apparent EC_50_ shift is observed most strongly only when cognate protease is co-expressed with cognate GPCR. Measurements done in biological triplicate after 8-h incubation. All experiments were run in biological triplicate and error bars represent the standard deviation.

**Figure S2. Protease’s promoter strength affects the extent of apparent EC_50_ shift.** Protease expression under lower strength promoters shifted the apparent EC_50_ less than higher strength promoters in all five tested proteases. Measurements done after 8-h incubation. All experiments were run in biological triplicate and error bars represent the standard deviation.

**Figure S3. Protease expression cassette copy number affects the extent of apparent EC_50_ shift.** One protease copy shifts apparent EC_50_ for about one order of magnitude in both ScSte2 and CaSte2 strains. Additional copy number marginally shifts the apparent EC_50_ towards higher peptide concentration. Measurements done after 8-h incubation. All experiments were run in biological triplicate and error bars represent the standard deviation.

**Figure S4. CaBar1 protease in biosensor strain with lycopene readout can effectively shift apparent EC_50_ of CaPep and CaPep13A, but not CaPep2A and CaPep2A13A.** Measurements done in biological triplicate after 24-h incubation. Error bars represent the standard deviation.


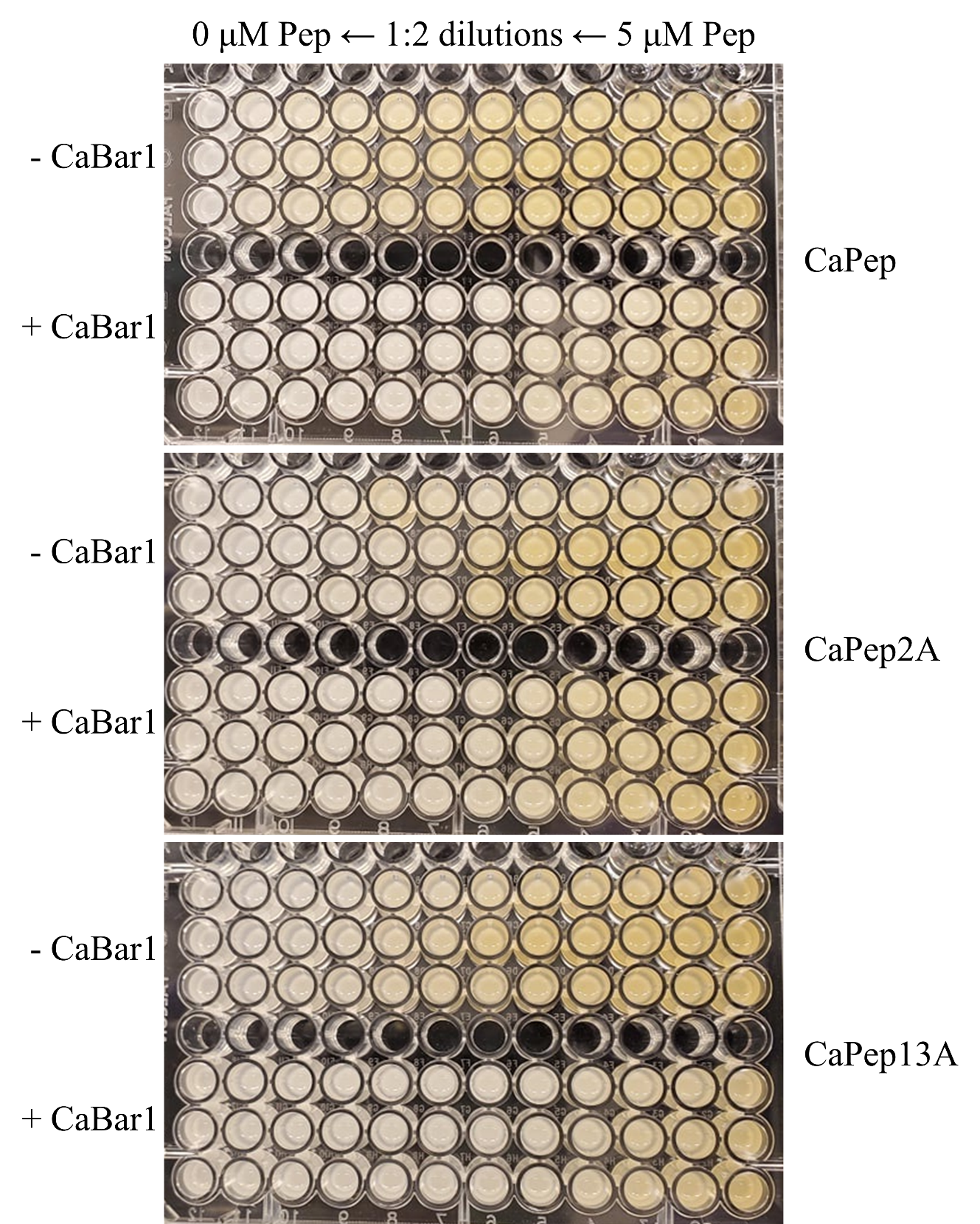


**Figure S5. CaBar1 protease in biosensor strain with lycopene readout diminishes color onset of CaPep and CaPep13A, but does not diminish of CaPep2A at the same peptide concentrations in liquid culture.** Peptides were added to cells after 1-h incubation, then the measurements and photos were taken after 8-h incubation. All experiments were run in biological triplicate.

**
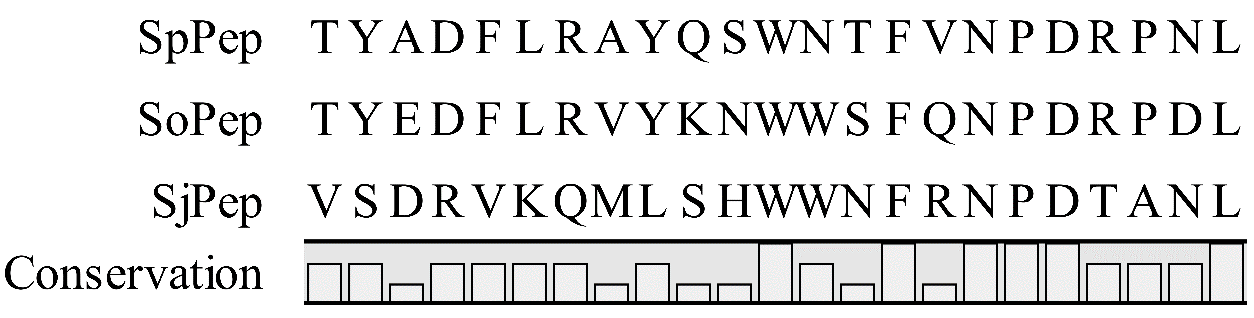
**

**Figure S6. SpPep, SoPep and SjPep alignment shows high sequence homology.**

**Table S1.** Strains used in this study.

| **Strain name** | **Genotype** | **Comment** | **Reference** |
| --- | --- | --- | --- |
| yWS890 | yWS677 *pCCW12-STE2-tSSA1-pPGK1-GPA1-tENO2-pRAD27-LexA-PRD-tENO1-URA3, LexO (6x)-pLEU2m-sfGFP-tTDH1-LEU2* | Starting strain for optimized parental strain | [1] |
| yTC363 | yWS890 *pCCW12-STE2-tSSA1 Δ* | Intermediary optimized parental strain | This study |
| yTC364 | yTC363 *URA3 Δ* | Intermediary optimized parental strain | This study |
| yTC370 | yTC364 *LexO (6x)-pLEU2m-sfGFP-tTDH1-LEU2 Δ* | Final optimized parental strain | This study |
| yTC391 | yTC370 *ARS208a:: pTDH3-ScSTE2-tSTE2-LexO(6x)-pLEU2m-ymTurquoise2-tTDH1-LEU2* | Integrated ScSte2 GPCR with ymTurquoise2 fluorescent readout | This study |
| yTC398 | yTC370 *ARS208a:: pTDH3-ScSTE2-tSTE2-LexO(6x)-pLEU2m-ymTurquoise2-tTDH1-pCCW12-ScBAR1-tENO2-LEU2* | Integrated ScSte2 GPCR with ymTurquoise2 fluorescent readout and ScBar1 protease | This study |
| yTC492 | yTC370 *ARS208a:: pTDH3-ScSTE2-tSTE2-LexO(6x)-pLEU2m-ymTurquoise2-tTDH1-pCCW12-CaBAR1-tENO2-LEU2* | Integrated ScSte2 GPCR with ymTurquoise2 fluorescent readout and CaBar1 protease | This study |
| yTC493 | yTC370 *ARS208a:: pTDH3-ScSTE2-tSTE2-LexO(6x)-pLEU2m-ymTurquoise2-tTDH1-pCCW12-SpSXA2-tENO2-LEU2* | Integrated ScSte2 GPCR with ymTurquoise2 fluorescent readout and SpSxa2 protease | This study |
| yTC494 | yTC370 *ARS208a:: pTDH3-ScSTE2-tSTE2-LexO(6x)-pLEU2m-ymTurquoise2-tTDH1-pCCW12- SjSXA2-tENO2-LEU2* | Integrated ScSte2 GPCR with ymTurquoise2 fluorescent readout and SjSxa2 protease | This study |
| yTC495 | yTC370 *ARS208a:: pTDH3-ScSTE2-tSTE2-LexO(6x)-pLEU2m-ymTurquoise2-tTDH1-pCCW12- SoSXA2-tENO2-LEU2* | Integrated ScSte2 GPCR with ymTurquoise2 fluorescent readout and SoSxa2 protease | This study |
| yTC384 | yTC370 *ARS208a:: pTDH3-CaSTE2-tSTE2-LexO(6x)-pLEU2m-ymTurquoise2-tTDH1-LEU2* | Integrated CaSte2 GPCR with ymTurquoise2 fluorescent readout | This study |
| yTC399 | yTC370 *ARS208a:: pTDH3-CaSTE2-tSTE2-LexO(6x)-pLEU2m-ymTurquoise2-tTDH1-pCCW12-CaBAR1-tENO2-LEU2* | Integrated CaSte2 GPCR with ymTurquoise2 fluorescent readout and CaBar1 protease | This study |
| yTC496 | yTC370 *ARS208a:: pTDH3-CaSTE2-tSTE2-LexO(6x)-pLEU2m-ymTurquoise2-tTDH1-pCCW12-ScBAR1-tENO2-LEU2* | Integrated CaSte2 GPCR with ymTurquoise2 fluorescent readout and ScBar1 protease | This study |
| yTC497 | yTC370 *ARS208a:: pTDH3-CaSTE2-tSTE2-LexO(6x)-pLEU2m-ymTurquoise2-tTDH1-pCCW12-SpSXA2-tENO2-LEU2* | Integrated CaSte2 GPCR with ymTurquoise2 fluorescent readout and SpSxa2 protease | This study |
| yTC498 | yTC370 *ARS208a:: pTDH3-CaSTE2-tSTE2-LexO(6x)-pLEU2m-ymTurquoise2-tTDH1-pCCW12- SjSXA2-tENO2-LEU2* | Integrated CaSte2 GPCR with ymTurquoise2 fluorescent readout and SjSxa2 protease | This study |
| yTC499 | yTC370 *ARS208a:: pTDH3-CaSTE2-tSTE2-LexO(6x)-pLEU2m-ymTurquoise2-tTDH1-pCCW12- SoSXA2-tENO2-LEU2* | Integrated CaSte2 GPCR with ymTurquoise2 fluorescent readout and SoSxa2 protease | This study |
| yTC530 | yTC370 *ARS208a:: pTDH3-SpSTE2-tSTE2-LexO(6x)-pLEU2m-ymTurquoise2-tTDH1-LEU2* *HO:: pTDH3-SpSTE2-tSTE2-LexO(6x)-pLEU2m-ymTurquoise2-tTDH1-HIS3* | Integrated SpSte2 GPCR with ymTurquoise2 fluorescent readout | This study |
| yTC529 | yTC370 *ARS208a:: pTDH3-SpSTE2-tSTE2-LexO(6x)-pLEU2m-ymTurquoise2-tTDH1-pCCW12-SpSXA2-tENO2-LEU2* *HO:: pTDH3-SpSTE2-tSTE2-LexO(6x)-pLEU2m-ymTurquoise2-tTDH1-pCCW12-SpSXA2-tENO2-HIS3* | Integrated SpSte2 GPCR with ymTurquoise2 fluorescent readout and SpSxa2 protease | This study |
| yTC533 | yTC370 *ARS208a:: pTDH3-SpSTE2-tSTE2-LexO(6x)-pLEU2m-ymTurquoise2-tTDH1-pCCW12-SjSXA2-tENO2-LEU2* *HO:: pTDH3-SpSTE2-tSTE2-LexO(6x)-pLEU2m-ymTurquoise2-tTDH1-pCCW12-SjSXA2-tENO2-HIS3* | Integrated SpSte2 GPCR with ymTurquoise2 fluorescent readout and SjSxa2 protease | This study |
| yTC534 | yTC370 *ARS208a:: pTDH3-SpSTE2-tSTE2-LexO(6x)-pLEU2m-ymTurquoise2-tTDH1-pCCW12-SoSXA2-tENO2-LEU2* *HO:: pTDH3-SpSTE2-tSTE2-LexO(6x)-pLEU2m-ymTurquoise2-tTDH1-pCCW12-SoSXA2-tENO2-HIS3* | Integrated SpSte2 GPCR with ymTurquoise2 fluorescent readout and SoSxa2 protease | This study |
| yTC531 | yTC370 *ARS208a:: pTDH3-SpSTE2-tSTE2-LexO(6x)-pLEU2m-ymTurquoise2-tTDH1-pCCW12-ScBAR1-tENO2-LEU2* *HO:: pTDH3-SpSTE2-tSTE2-LexO(6x)-pLEU2m-ymTurquoise2-tTDH1-pCCW12-ScBAR1-tENO2-HIS3* | Integrated SpSte2 GPCR with ymTurquoise2 fluorescent readout and ScBar1 protease | This study |
| yTC532 | yTC370 *ARS208a:: pTDH3-SpSTE2-tSTE2-LexO(6x)-pLEU2m-ymTurquoise2-tTDH1-pCCW12-CaBAR1-tENO2-LEU2* *HO:: pTDH3-SpSTE2-tSTE2-LexO(6x)-pLEU2m-ymTurquoise2-tTDH1-pCCW12-CaBAR1-tENO2-HIS3* | Integrated SpSte2 GPCR with ymTurquoise2 fluorescent readout and CaBar1 protease | This study |
| yTC404 | yTC370 *ARS208a:: pTDH3-SjSTE2-tSTE2-LexO(6x)-pLEU2m-ymTurquoise2-tTDH1-LEU2* | Integrated SjSte2 GPCR with ymTurquoise2 fluorescent readout | This study |
| yTC401 | yTC370 *ARS208a:: pTDH3-SjSTE2-tSTE2-LexO(6x)-pLEU2m-ymTurquoise2-tTDH1-pCCW12-SjSXA2-tENO2-LEU2* | Integrated SjSte2 GPCR with ymTurquoise2 fluorescent readout and SjSxa2 protease | This study |
| yTC507 | yTC370 *ARS208a:: pTDH3-SjSTE2-tSTE2-LexO(6x)-pLEU2m-ymTurquoise2-tTDH1-pCCW12-SoSXA2-tENO2-LEU2* | Integrated SjSte2 GPCR with ymTurquoise2 fluorescent readout and SoSxa2 protease | This study |
| yTC504 | yTC370 *ARS208a:: pTDH3-SjSTE2-tSTE2-LexO(6x)-pLEU2m-ymTurquoise2-tTDH1-pCCW12-ScBAR1-tENO2-LEU2* | Integrated SjSte2 GPCR with ymTurquoise2 fluorescent readout and ScBar1 protease | This study |
| yTC505 | yTC370 *ARS208a:: pTDH3-SjSTE2-tSTE2-LexO(6x)-pLEU2m-ymTurquoise2-tTDH1-pCCW12-CaBAR1-tENO2-LEU2* | Integrated SjSte2 GPCR with ymTurquoise2 fluorescent readout and CaBar1 protease | This study |
| yTC506 | yTC370 *ARS208a:: pTDH3-SjSTE2-tSTE2-LexO(6x)-pLEU2m-ymTurquoise2-tTDH1-pCCW12-SpSXA2-tENO2-LEU2* | Integrated SjSte2 GPCR with ymTurquoise2 fluorescent readout and SpSxa2 protease | This study |
| yTC405 | yTC370 *ARS208a:: pTDH3-SoSTE2-tSTE2-LexO(6x)-pLEU2m-ymTurquoise2-tTDH1-LEU2* | Integrated SoSte2 GPCR with ymTurquoise2 fluorescent readout | This study |
| yTC402 | yTC370 *ARS208a:: pTDH3-SoSTE2-tSTE2-LexO(6x)-pLEU2m-ymTurquoise2-tTDH1-pCCW12-SoSXA2-tENO2-LEU2* | Integrated SoSte2 GPCR with ymTurquoise2 fluorescent readout and SoSxa2 protease | This study |
| yTC508 | yTC370 *ARS208a:: pTDH3-SoSTE2-tSTE2-LexO(6x)-pLEU2m-ymTurquoise2-tTDH1-pCCW12-ScBAR1-tENO2-LEU2* | Integrated SoSte2 GPCR with ymTurquoise2 fluorescent readout and ScBar1 protease | This study |
| yTC509 | yTC370 *ARS208a:: pTDH3-SoSTE2-tSTE2-LexO(6x)-pLEU2m-ymTurquoise2-tTDH1-pCCW12-CaBAR1-tENO2-LEU2* | Integrated SoSte2 GPCR with ymTurquoise2 fluorescent readout and CaBar1 protease | This study |
| yTC510 | yTC370 *ARS208a:: pTDH3-SoSTE2-tSTE2-LexO(6x)-pLEU2m-ymTurquoise2-tTDH1-pCCW12-SpSXA2-tENO2-LEU2* | Integrated SoSte2 GPCR with ymTurquoise2 fluorescent readout and SpSxa2 protease | This study |
| yTC511 | yTC370 *ARS208a:: pTDH3-SoSTE2-tSTE2-LexO(6x)-pLEU2m-ymTurquoise2-tTDH1-pCCW12-SjSXA2-tENO2-LEU2* | Integrated SoSte2 GPCR with ymTurquoise2 fluorescent readout and SjSxa2 protease | This study |
| yTC512 | yTC370 *ARS208a:: pTDH3-ScSTE2-tSTE2-LexO(6x)-pLEU2m-ymTurquoise2-tTDH1-pRPL18B-ScBAR1-tENO2-LEU2* | Integrated ScSte2 GPCR with ymTurquoise2 fluorescent readout and ScBar1 protease with medium expression level promoter | This study |
| yTC513 | yTC370 *ARS208a:: pTDH3-CaSTE2-tSTE2-LexO(6x)-pLEU2m-ymTurquoise2-tTDH1-pRPL18B-CaBAR1-tENO2-LEU2* | Integrated CaSte2 GPCR with ymTurquoise2 fluorescent readout and CaBar1 protease with medium expression level promoter | This study |
| yTC525 | yTC370 *ARS208a:: pTDH3-SpSTE2-tSTE2-LexO(6x)-pLEU2m-ymTurquoise2-tTDH1-pRPL18B-SpSXA2-tENO2-LEU2 HO:: pTDH3-SpSTE2-tSTE2-LexO(6x)-pLEU2m-ymTurquoise2-tTDH1-pRPL18B-SpSXA2-tENO2-LEU2* | Integrated SpSte2 GPCR with ymTurquoise2 fluorescent readout and SpSxa2 protease with medium expression level promoter | This study |
| yTC515 | yTC370 *ARS208a:: pTDH3-SjSTE2-tSTE2-LexO(6x)-pLEU2m-ymTurquoise2-tTDH1-pRPL18B-SjSXA2-tENO2-LEU2* | Integrated SjSte2 GPCR with ymTurquoise2 fluorescent readout and SjSxa2 protease with medium expression level promoter | This study |
| yTC516 | yTC370 *ARS208a:: pTDH3-SoSTE2-tSTE2-LexO(6x)-pLEU2m-ymTurquoise2-tTDH1-pRPL18B-SoSXA2-tENO2-LEU2* | Integrated SoSte2 GPCR with ymTurquoise2 fluorescent readout and SoSxa2 protease with medium expression level promoter | This study |
| yTC517 | yTC370 *ARS208a:: pTDH3-ScSTE2-tSTE2-LexO(6x)-pLEU2m-ymTurquoise2-tTDH1-pRAD27-ScBAR1-tENO2-LEU2* | Integrated ScSte2 GPCR with ymTurquoise2 fluorescent readout and ScBar1 protease with low expression level promoter | This study |
| yTC518 | yTC370 *ARS208a:: pTDH3-CaSTE2-tSTE2-LexO(6x)-pLEU2m-ymTurquoise2-tTDH1-pRAD27-CaBAR1-tENO2-LEU2* | Integrated CaSte2 GPCR with ymTurquoise2 fluorescent readout and CaBar1 protease with low expression level promoter | This study |
| yTC526 | yTC370 *ARS208a:: pTDH3-SpSTE2-tSTE2-LexO(6x)-pLEU2m-ymTurquoise2-tTDH1-pRAD27-SpSXA2-tENO2-LEU2 HO:: pTDH3-SpSTE2-tSTE2-LexO(6x)-pLEU2m-ymTurquoise2-tTDH1-pRAD27-SpSXA2-tENO2-LEU2* | Integrated SpSte2 GPCR with ymTurquoise2 fluorescent readout and SpSxa2 protease with low expression level promoter | This study |
| yTC520 | yTC370 *ARS208a:: pTDH3-SjSTE2-tSTE2-LexO(6x)-pLEU2m-ymTurquoise2-tTDH1-pRAD27-SjSXA2-tENO2-LEU2* | Integrated SjSte2 GPCR with ymTurquoise2 fluorescent readout and SjSxa2 protease with low expression level promoter | This study |
| yTC521 | yTC370 *ARS208a:: pTDH3-SoSTE2-tSTE2-LexO(6x)-pLEU2m-ymTurquoise2-tTDH1-pRAD27-SoSxa2-tENO2-LEU2* | Integrated SoSte2 GPCR with ymTurquoise2 fluorescent readout and SoSxa2 protease with low expression level promoter | This study |
| yBB01 | yTC370 *ARS208a:: pTDH3-ScSTE2-tSTE2-LexO(6x)-pLEU2m-ymTurquoise2-tTDH1-pCCW12-ScBAR1-tENO2-LEU2* *HO:: pCCW12-ScBAR1-tENO2-HIS3* | Integrated ScSte2 GPCR with ymTurquoise2 fluorescent readout and ScBar1 protease (two copies) | This study |
| yBB02 | yTC370 *ARS208a:: pTDH3-CaSTE2-tSTE2-LexO(6x)-pLEU2m-ymTurquoise2-tTDH1-pCCW12-CaBAR1-tENO2-LEU2* *HO:: pCCW12-CaBAR1-tENO2-HIS3* | Integrated CaSte2 GPCR with ymTurquoise2 fluorescent readout and CaBar1 protease (two copies) | This study |
| yTC412 | yTC370 *ARS208a:: pTDH3-FAD1-tPGK1-LexO(6x)-pLEU2m-CrtI-tACT1-LEU2 HO:: pTEF1-CrtE-tADH1-CrtB-pPGK1-LexO(6x)-pLEU2m-CrtI-tACT1-HIS3* | Receptor-less lycopene strain with two pheromone-inducible copies of CrtI | This study |
| yTC681 | yTC370 *ARS208a:: pTDH3-FAD1-tPGK1-LexO(6x)-pLEU2m-CrtI-tACT1-pCCW12-CaBAR1-tENO2-LEU2 HO:: pTEF1-CrtE-tADH1-CrtB-pPGK1-LexO(6x)-pLEU2m-CrtI-tACT1-HIS3* | Receptor-less lycopene strain with two pheromone-inducible copies of CrtI and constitutive CaBar1 protease | This study |
| yTC646 | yTC412 *LEU2:: pTDH3-CaSTE2-tSTE2-URA3* | CaSte2 lycopene biosensor | This study |
| yTC682 | yTC681 *LEU2:: pTDH3-CaSTE2-tSTE2-pCCW12-CaBAR1-tENO2-URA3* | CaSte2 + CaBar1 lycopene biosensor | This study |

**Table S2.** Plasmids used in this study. Plasmids were generated in this study except where a source is noted.

| **Name** | **Construct details** | **Selection** | **Comments** |
| --- | --- | --- | --- |
| pTC160 | Cas9-gRNA (STE2) | Amp, NAT | CRISPR genomic integration |
| pTC200 | Cas9-gRNA (URA3) | Amp, NAT | CRISPR genomic integration |
| pTC220 | Cas9-gRNA (LEU2) | Amp, U- | CRISPR genomic integration |
| pTC11 | Cas9-gRNA (ARS208a) | Amp, U- | CRISPR genomic integration |
| pTC12 | Cas9-gRNA (HO) | Amp, U- | CRISPR genomic integration |
| pTC393 | LEU2_CAGT_RFP_TTTT_URA3 | Amp | Acceptor plasmid in GGA |
| pTC197 | ARS208a_CAGT_RFP_TTTT_LEU2 | Amp | Acceptor plasmid in GGA |
| pTC199 | HO_CAGT_RFP_TTTT_HIS3 | Amp | Acceptor plasmid in GGA |
| pTC394 | Cas9-gRNA (LEU2) | Amp, NAT | CRISPR genomic integration |
| pTC223 | CAGT-pGPD_CaSTE2_tSTE2-CCAA | Cam | GPCR cassette plasmid |
| pTC230 | CAGT-pGPD_ScSTE2_tSTE2-CCAA | Cam | GPCR cassette plasmid |
| pTC231 | CAGT-pGPD_SjSTE2_tSTE2-CCAA | Cam | GPCR cassette plasmid |
| pTC232 | CAGT-pGPD_SoSTE2_tSTE2-CCAA | Cam | GPCR cassette plasmid |
| pTC233 | CAGT-pGPD_SpSTE2_tSTE2-CCAA | Cam | GPCR cassette plasmid |
| pTC239 | CCAA-LexO(6x)-pLEU2m-ymTurquoise2_tTDH1-GCAT | Cam | Fluorescent readout cassette plasmid |
| pTC262 | GAGT-pCCW12_ScBAR1_tENO2-TTTT | Cam | Protease cassette plasmid |
| pTC263 | GAGT-pCCW12_CaBAR1_tENO2-TTTT | Cam | Protease cassette plasmid |
| pTC264 | GAGT-pCCW12_SpSXA2_tENO2-TTTT | Cam | Protease cassette plasmid |
| pTC265 | GAGT-pCCW12_SjSXA2_tENO2-TTTT | Cam | Protease cassette plasmid |
| pTC266 | GAGT-pCCW12_SoSXA2_tENO2-TTTT | Cam | Protease cassette plasmid |
| pTC355 | GAGT-pRPL18B_ScBAR1_tENO2-TTTT | Cam | Protease cassette plasmid |
| pTC356 | GAGT-pRPL18B_CaBAR1_tENO2-TTTT | Cam | Protease cassette plasmid |
| pTC357 | GAGT-pRPL18B_SpSXA2_tENO2-TTTT | Cam | Protease cassette plasmid |
| pTC358 | GAGT-pRPL18B_SjSXA2_tENO2-TTTT | Cam | Protease cassette plasmid |
| pTC359 | GAGT-pRPL18B_SoSXA2_tENO2-TTTT | Cam | Protease cassette plasmid |
| pTC360 | GAGT-pRAD27_ScBAR1_tENO2-TTTT | Cam | Protease cassette plasmid |
| pTC361 | GAGT-pRAD27_CaBAR1_tENO2-TTTT | Cam | Protease cassette plasmid |
| pTC362 | GAGT-pRAD27_SpSXA2_tENO2-TTTT | Cam | Protease cassette plasmid |
| pTC363 | GAGT-pRAD27_SjSXA2_tENO2-TTTT | Cam | Protease cassette plasmid |
| pTC364 | GAGT-pRAD27_SoSXA2_tENO2-TTTT | Cam | Protease cassette plasmid |
| pTC288 | CCAA_GCAT | Cam | Connector cassette plasmid |
| pTC289 | GCAT_GAGT | Cam | Connector cassette plasmid |
| pTC290 | GAGT_TTTT | Cam | Connector cassette plasmid |
| pTC291 | GCAT_TTTT | Cam | Connector cassette plasmid |
| pTC274 | ARS208a_CaSTE2_ymTurquoise2_LEU2 | Amp | Complete integration plasmid |
| pTC281 | ARS208a_ScSTE2_ymTurquoise2_LEU2 | Amp | Complete integration plasmid |
| pTC292 | ARS208a_ScSTE2_ymTurquoise2_ScBAR1_LEU2 | Amp | Complete integration plasmid |
| pTC293 | ARS208a_CaSTE2_ymTurquoise2_CaBAR1_LEU2 | Amp | Complete integration plasmid |
| pTC294 | ARS208a_SpSTE2_ymTurquoise2_SpSXA2_LEU2 | Amp | Complete integration plasmid |
| pTC295 | ARS208a_SjSTE2_ymTurquoise2_SjSXA2_LEU2 | Amp | Complete integration plasmid |
| pTC296 | ARS208a_SoSTE2_ymTurquoise2_SoSXA2_LEU2 | Amp | Complete integration plasmid |
| pTC297 | ARS208a_SpSTE2_ymTurquoise2_LEU2 | Amp | Complete integration plasmid |
| pTC298 | ARS208a_SjSTE2_ymTurquoise2_LEU2 | Amp | Complete integration plasmid |
| pTC299 | ARS208a_SoSTE2_ymTurquoise2_LEU2 | Amp | Complete integration plasmid |
| pTC300 | HO_CrtE_CrtB_LexO-CrtI_HIS3 | Amp | Complete integration plasmid |
| pTC301 | ARS208a_FAD1_LexO-CrtI_LEU2 | Amp | Complete integration plasmid |
| pTC335 | ARS208a_ScSTE2_ymTurquoise2_CaBAR1_LEU2 | Amp | Complete integration plasmid |
| pTC336 | ARS208a_ScSTE2_ymTurquoise2_SpSXA2_LEU2 | Amp | Complete integration plasmid |
| pTC337 | ARS208a_ScSTE2_ymTurquoise2_SjSXA2_LEU2 | Amp | Complete integration plasmid |
| pTC338 | ARS208a_ScSTE2_ymTurquoise2_SoSXA2_LEU2 | Amp | Complete integration plasmid |
| pTC339 | ARS208a_CaSTE2_ymTurquoise2_ScBAR1_LEU2 | Amp | Complete integration plasmid |
| pTC340 | ARS208a_CaSTE2_ymTurquoise2_SpSXA2_LEU2 | Amp | Complete integration plasmid |
| pTC341 | ARS208a_CaSTE2_ymTurquoise2_SjSXA2_LEU2 | Amp | Complete integration plasmid |
| pTC342 | ARS208a_CaSTE2_ymTurquoise2_SoSXA2_LEU2 | Amp | Complete integration plasmid |
| pTC343 | ARS208a_SpSTE2_ymTurquoise2_Sc BAR1_LEU2 | Amp | Complete integration plasmid |
| pTC344 | ARS208a_SpSTE2_ymTurquoise2_Ca BAR1_LEU2 | Amp | Complete integration plasmid |
| pTC345 | ARS208a_SpSTE2_ymTurquoise2_SjSXA2_LEU2 | Amp | Complete integration plasmid |
| pTC346 | ARS208a_SpSTE2_ymTurquoise2_SoSXA2_LEU2 | Amp | Complete integration plasmid |
| pTC347 | ARS208a_SjSTE2_ymTurquoise2_Sc BAR1_LEU2 | Amp | Complete integration plasmid |
| pTC348 | ARS208a_SjSTE2_ymTurquoise2_Ca BAR1_LEU2 | Amp | Complete integration plasmid |
| pTC349 | ARS208a_SjSTE2_ymTurquoise2_SpSXA2_LEU2 | Amp | Complete integration plasmid |
| pTC350 | ARS208a_SjSTE2_ymTurquoise2_SoSXA2_LEU2 | Amp | Complete integration plasmid |
| pTC351 | ARS208a_SoSTE2_ymTurquoise2_Sc BAR1_LEU2 | Amp | Complete integration plasmid |
| pTC352 | ARS208a_SoSTE2_ymTurquoise2_Ca BAR1_LEU2 | Amp | Complete integration plasmid |
| pTC353 | ARS208a_SoSTE2_ymTurquoise2_SpSXA2_LEU2 | Amp | Complete integration plasmid |
| pTC354 | ARS208a_SoSTE2_ymTurquoise2_SjSXA2_LEU2 | Amp | Complete integration plasmid |
| pTC368 | HO_SpSTE2_ymTurquoise2_SpSXA2_HIS3 | Amp | Complete integration plasmid |
| pTC369 | HO_SpSTE2_ymTurquoise2_HIS3 | Amp | Complete integration plasmid |
| pTC399 | ARS208a_ScSTE2_ymTurquoise2_ScBAR1 (med)_LEU2 | Amp | Complete integration plasmid |
| pTC400 | ARS208a_CaSTE2_ymTurquoise2_CaBAR1 (med)_LEU2 | Amp | Complete integration plasmid |
| pTC401 | ARS208a_SpSTE2_ymTurquoise2_SpSXA2 (med)_LEU2 | Amp | Complete integration plasmid |
| pTC402 | ARS208a_SjSTE2_ymTurquoise2_SjSXA2 (med)_LEU2 | Amp | Complete integration plasmid |
| pTC403 | ARS208a_SoSTE2_ymTurquoise2_SoSXA2 (med)_LEU2 | Amp | Complete integration plasmid |
| pTC404 | ARS208a_ScSTE2_ymTurquoise2_ScBAR1 (low)_LEU2 | Amp | Complete integration plasmid |
| pTC405 | ARS208a_CaSTE2_ymTurquoise2_CaBAR1 (low)_LEU2 | Amp | Complete integration plasmid |
| pTC406 | ARS208a_SpSTE2_ymTurquoise2_SpSXA2 (low)_LEU2 | Amp | Complete integration plasmid |
| pTC407 | ARS208a_SjSTE2_ymTurquoise2_SjSXA2 (low)_LEU2 | Amp | Complete integration plasmid |
| pTC408 | ARS208a_SoSTE2_ymTurquoise2_SoSXA2 (low)_LEU2 | Amp | Complete integration plasmid |
| pTC420 | HO_SpSTE2_ymTurquoise2_ScBAR1_HIS3 | Amp | Complete integration plasmid |
| pTC421 | HO_SpSTE2_ymTurquoise2_CaBAR1_HIS3 | Amp | Complete integration plasmid |
| pTC422 | HO_SpSTE2_ymTurquoise2_SjSXA2_HIS3 | Amp | Complete integration plasmid |
| pTC423 | HO_SpSTE2_ymTurquoise2_SoSXA2_HIS3 | Amp | Complete integration plasmid |
| pTC424 | HO_SpSTE2_ymTurquoise2_SpSXA2 (med)_HIS3 | Amp | Complete integration plasmid |
| pTC425 | HO_SpSTE2_ymTurquoise2_SpSXA2 (low)_HIS3 | Amp | Complete integration plasmid |
| pBB01 | HO_ScBAR1_HIS3 | Amp | Complete integration plasmid |
| pBB02 | HO_CaBAR1_HIS3 | Amp | Complete integration plasmid |
| pTC546 | LEU2_CaSTE2_CaBAR1_URA3 | Amp | Complete integration plasmid |
| pTC549 | LEU2_CaSTE2_URA3 | Amp | Complete integration plasmid |
| pTC561 | ARS208a_FAD1_LexO_CrtI_CaBAR1_LEU2 | Amp | Complete integration plasmid |

**Table S3.** List of expression modules and cassette sequences constructed in this study. Promoters and terminators are in upper case, open reading frames (ORFs) in lower case. BsaI restriction enzyme recognition site is highlighted grey. Golden Gate Assembly 4-bp overlaps are in **bold**.

| **Description** | **Sequence** |
| --- | --- |
| GPCR cassette sequence | |
| pTDH3-  GPCR-  tSTE2 | GGTCTCA**CAGT**AGTTTATCATTATCAATACTGCCATTTCAAAGAATACGTAAATAATTAATAGTAGTGATTTTCCTAACTTTATTTAGTCAAAAAATTAGCCTTTTAATTCTGCTGTAACCCGTACATGCCCAAAATAGGGGGCGGGTTACACAGAATATATAACATCGTAGGTGTCTGGGTGAACAGTTTATTCCTGGCATCCACTAAATATAATGGAGCCCGCTTTTTAAGCTGGCATCCAGAAAAAAAAAGAATCCCAGCACCAAAATATTGTTTTCTTCACCAACCATCAGTTCATAGGTCCATTCTCTTAGCGCAACTACAGAGAACAGGGGCACAAACAGGCAAAAAACGGGCACAACCTCAATGGAGTGATGCAACCTGCCTGGAGTAAATGATGACACAAGGCAATTGACCCACGCATGTATCTATCTCATTTTCTTACACCTTCTATTACCTTCTGCTCTCTCTGATTTGGAAAAAGCTGAAAAAAAAGGTTGAAACCAGTTCCCTGAAATTATTCCCCTACTTGACTAATAAGTATATAAAGACGGTAGGTATTGATTGTAATTCTGTAAATCTATTTCTTAAACTTCTTAAATTCTACTTTTATAGTTAGTCTTTTTTTTAGTTTTAAAACACCAAGAACTTAGTTTCGACGGATACTAGTAAA[receptor ORF]CTCGAGACGGCTTTGAAAAAGTAATTTCGTGACCTTCGGTATAAGGTTACTACTAGATTCAGGTGCTCATCAGATGCACCACATTCTCTATAAAAAAAAATGGTATCTTTCTTATTTGATAATATTTAAACTCCTTTACATAATAAACATCTCGTAAGTAGTGGTAGAAACCACCTTTGCTTTTACGAGTTCA**CCAA**CGAGACC |
| Readout cassette sequence | |
| LexO(6x)-pLEU2m-readout-tTDH1 | GGTCTCA**CCAA**AAACGGGTAGTCCATCGTTGTAGGATACTGTATATACACCCAGTAGAGTAGGTGACTACTGTATGAGCATACAGTAGTGACAACCACTTACTGTATATAAATACAGTAGTGGTCATCGATACTGTATATAAAACCAGTAGGCAATCGTTTGTACTGTATGTACATACAGTATACCTCGCAACTACTGTATATAAACACAGTAATTAACTTGTAATATTCTAATCAATTGACAATATTATTTAAGGACCTATTGTTTTTTCCAATAGGTGGTTAGCAATCGTCTTACTTTCTAACTTTTCTTACCTTTTACATTTCAGCAATATATATATATATATTTCAAGGATATACCATTCTAAGATCTG[readout ORF]CTAACTCGAGATAAAGCAATCTTGATGAGGATAATGATTTTTTTTTGAATATACATAAATACTACCGTTTTTCTGCTAGATTTTGTGATGACGTAAATAAGTACATATTACTTTTTAAGCCAAGACAAGATTAAGCATTAACTTTACCCTTTTCTTTCTAAGTTTCAATATTAGTTATCACTGTTTAAAAGTTATGGCGAGAACGTCGGCGGTTAAAATATATTACCCTGAACG**GCAT**TGAGACC |
| Peptide protease cassette sequence | |
| pCCW12-protease-tENO2 (high expression) | GGTCTCA**GAGT**CACCCATGAACCACACGGTTAGTCCAAAAGGGGCAGTTCAGATTCCAGATGCGGGAATTAGCTTGCTGCCACCCTCACCTCACTAACGCTGCGGTGTGCGGATACTTCATGCTATTTATAGACGCGCGTGTCGGAATCAGCACGCGCAAGAACCAAATGGGAAAATCGGAATGGGTCCAGAACTGCTTTGAGTGCTGGCTATTGGCGTCTGATTTCCGTTTTGGGAATCCTTTGCCGCGCGCCCCTCTCAAAACTCCGCACAAGTCCCAGAAAGCGGGAAAGAAATAAAACGCCACCAAAAAAAAAAAAATAAAAGCCAATCCTCGAAGCGTGGGTGGTAGGCCCTGGATTATCCCGTACAAGTATTTCTCAGGAGTAAAAAAACCGTTTGTTTTGGAATTTCCCATTTCGCGGCCACCTACGCCGCTATCTTTGCAACAACTATCTGCGATAACTCAGCAAATTTTGCATATTCGTGTTGCAGTATTGCGATAATGGGAGTCTTACTTCCAACATAACGGCAGAAAGAAATGTGAGAAAATTTTGCATCCTTTGCCTCCGTTCAAGTATATAAAGTCGGCATGCTTGATAATCTTTCTTTCCATCCTACATTGTTCTAATTATTCTTATTCTCCTTTATTCTTTCCTAACATACCAAGAAATTAATCTTCTGTCATTCGCTTAAACACTATATCAATAAAGATCACTAGT [protease ORF]ACTAGTTAACTCGAGAGTGCTTTTAACTAAGAATTATTAGTCTTTTCTGCTTATTTTTTCATCATAGTTTAGAACACTTTATATTAACGAATAGTTTATGAATCTATTTAGGTTTAAAAATTGATACAGTTTTATAAGTTACTTTTTCAAAGACTCGTGCTGTCTATTGCATAATGCACTGGAAGGGGAAAAAAAAGGTGCACACGCGTGGCTTTTTCTTGAATTTGCAGTTTGAAAAAT**TTTT**TGAGACC |
| pRPL18B-protease-tENO2 (medium expression) | GGTCTCA**GAGT**AAGAGGATGTCCAATATTTTTTTTAAGGAATAAGGATACTTCAAGACTAGATTCCCCCCTGCATTCCCATCAGAACCGTAAACCTTGGCGCTTTCCTTGGGAAGTATTCAAGAAGTGCCTTGTCCGGTTTCTGTGGCTCACAAACCAGCGCGCCCGATATGGCTTTCTTTTCACTTATGAATGTACCAGTACGGGACAATTAGAACGCTCCTGTAACAATCTCTTTGCAAATGTGGGGTTACATTCTAACCATGTCACACTGCTGACGAAATTCAAAGTAAAAAAAAATGGGACCACGTCTTGAGAACGATAGATTTTCTTTATTTTACATTGAACAGTCGTTGTCTCAGCGCGCTTTATGTTTTCATTCATACTTCATATTATAAAATAACAAAAGAAGAATTTCATATTCACGCCCAAGAAATCAGGCTGCTTTCCAAATGCAATTGACACTTCATTAGCCATCACACAAAACTCTTTCTTGCTGGAGCTTCTTTTAAAAAAGACCTCAGTACACCAAACACGTTACCCGACCTCGTTATTTTACGACAACTATGATAAAATTCTGAAGAAAAAATAAAAAAATTTTCATACTTCTTGCTTTTATTTAAACCATTGAATGATTTCTTTTGAACAAAACTACCTGTTTCACCAAAGGAAATAGAAAGAAAAAATCAATTAGAAGAAAACAAAAAACAAAAGATCT[protease ORF]ACTAGTTAACTCGAGAGTGCTTTTAACTAAGAATTATTAGTCTTTTCTGCTTATTTTTTCATCATAGTTTAGAACACTTTATATTAACGAATAGTTTATGAATCTATTTAGGTTTAAAAATTGATACAGTTTTATAAGTTACTTTTTCAAAGACTCGTGCTGTCTATTGCATAATGCACTGGAAGGGGAAAAAAAAGGTGCACACGCGTGGCTTTTTCTTGAATTTGCAGTTTGAAAAAT**TTTT**TGAGACC |
| pRAD27-protease-tENO2 (low expression) | GGTCTCA**GAGT**CCTTGTGAAATTGCAAATATGGTGATTTGAAACGTTTCCTAGTGCAGCAGGATCACAGATAACGTGTAAAGGGCTTAGCAGTTGATAATCCTCTCTAGTTAAGACCTAAACAAAATGCTGTCACTAACCGTAGTATTAAATGACACACTTTGGTGACTTTCGTTAATGGGGATGTGGTAGTGGCCATTGCCAATAAACAAAAAGAACAGGGAAAGAAGTAGAAAGTGATATAAGTTTGCTTGCCACTTTTCGTTTTTCACGAAAAAAACAGGCGAAAAAAAATGCTAGACAAGTACCCGGCTGAATCACACCTCGTTAACAGTGACTTTCGGTGACAGATACCCGATTGGGCACCCGGCTGGTAAGTTATGATAGAAAGCCAACGCTGTACTATTGGCTTAGCTATGGCAATATTTTGATTATCAGCTAGTTTTATTAACGTTATAATTAGTGTAACCAGTTTTTCATCTATTTCATTTATTTCATTTATTTACTTTAATTGCAGATCCCCCTAACGCGTTTAAAGCTTTTATTCACTAGCTTATGTATTTTTTATAGGAAACGCGACGCGTAACATCGCGCAAATGAAGGTTTTGATGTATTATAATGAGGTATTCTTCCTTATATACATCGATGAAAAGCGTTGACAGCATACATTGGAAAGAAATAGGAAACGGACACCGGAAGAAAAAATAGATCT[protease ORF]ACTAGTTAACTCGAGAGTGCTTTTAACTAAGAATTATTAGTCTTTTCTGCTTATTTTTTCATCATAGTTTAGAACACTTTATATTAACGAATAGTTTATGAATCTATTTAGGTTTAAAAATTGATACAGTTTTATAAGTTACTTTTTCAAAGACTCGTGCTGTCTATTGCATAATGCACTGGAAGGGGAAAAAAAAGGTGCACACGCGTGGCTTTTTCTTGAATTTGCAGTTTGAAAAAT**TTTT**TGAGACC |
| Connecting part sequences | |
| CCAA-GCAT | GGTCTCA**CCAA**GCGCCGGGC**GCAT**TGAGACC |
| GCAT-GAGT | GGTCTCA**GCAT**GCGCCGGGC**GAGT**TGAGACC |
| GAGT-TTTT | GGTCTCA**GAGT**GCGCCGGGC**TTTT**TGAGACC |
| GCAT-TTTT | GGTCTCA**GCAT**GCGCCGGGC**TTTT**TGAGACC |
| Lycopene cassette sequences | |
| pTEF1-  CrtE-tADH1-  (CrtB-pPGK1,  rev), LexO(6x)-pLEU2m-CrtI-tACT1 | GGTCTCA**CAGT**ATAGCTTCAAAATGTTTCTACTCCTTTTTTACTCTTCCAGATTTTCTCGGACTCCGCGCATCGCCGTACCACTTCAAAACACCCAAGCACAGCATACTAAATTTCCCCTCTTTCTTCCTCTAGGGTGTCGTTAATTACCCGTACTAAAGGTTTGGAAAAGAAAAAAGAGACCGCCTCGTTTCTTTTTCTTCGTCGAAAAAGGCAATAAAAATTTTTATCACGTTTCTTTTTCTTGAAAATTTTTTTTTTTGATTTTTTTCTCTTTCGATGACCTCCCATTGATATTTAAGTTAATAAACGGTCTTCAATTTCTCAAGTTTCAGTTTCATTTTTCTTGTTCTATTACAACTTTTTTTACTTCTTGCTCATTAGAAAGAAAGCATAGCAATCTAATCTAAGTTTTAATTACAAAatggtttctggttcgaaagcaggagtatcacctcatagggaaatcgaagtcatgagacagtccattgatgaccacttagcaggattgttgccagaaacagattcccaggatatcgttagccttgctatgagagaaggtgttatggcacctggtaaacgtatcagacctttgctgatgttacttgctgcaagGgacctgagatatcagggttctatgcctacactactggatctagcttgtgctgttgaactgacacatactgcttccttgatgctggatgacatgccttgtatggacaatgcggaacttagaagaggtcaaccaacaacccacaagaaattcggagaatctgttgccattttggcttctgtaggtctgttgtcgaaagcttttggcttgattgctgcaactggtgatcttccaggtgaaaggagagcacaagctgtaaacgagctatctactgcagttggtgttcaaggtctagtcttaggacagttcagagatttgaatgacgcagctttggacagaactcctgatgctatcctgtctacgaaccatctgaagactggcatcttgttctcagctatgttgcaaatcgtagccattgcttctgcttcttcaccatctactagggaaacgttacacgcattcgcattggactttggtcaagcctttcaactgctagacgatttgagggatgatcatccagagacaggtaaagaccgtaacaaagacgctggtaaaagcactctagtcaacagattgggtgctgatgcagctagacagaaactgagagagcacattgactctgctgacaaacacctgacatttgcatgtccacaaggaggtgctataaggcagtttatgcacctatggtttggacaccatcttgctgattggtcCccagtgatgaagatcgcctaaGCGAATTTCTTATGATTTATGATTTTTATTATTAAATAAGTTATAAAAAAAATAAGTGTATACAAATTTTAAAGTGACTCTTAGGTTTTAAAACGAAAATTCTTATTCTTGAGTAACTCTTTCCTGTAGGTCAGGTTGCTTTCTCAGGTATAGCATGAGGTCGCTCttagacaggtctttgccataaaccagcaggtcttggtgtaactctggttgttttggcacgaatgacttgtcctggtgcagccataagcatcgcaatcttctctcctttgctagtgtgttgacgtctatcccaagcagaaccacctgcagcttttaccttgatgccaatctctctgtagacagatcttgcagtagctatagcccaagcacatctaggtggtagatcatgcaatccagcttgacttgagatgtaatagggttcagcagcgtctatcagcctttcagcaactcttgctaatgcagctctgttctctcttgcagcgtaattctcaggagttagaccagcatcttgcaaccattcagcaggtagatagcatctgtcaatagctgcatcgtcgataatatctctcgcgatgtttgtcagctgaaaagccaaacctagatcacaagctctgtccaaaaccctttcgtctcttacacccattactcttgccatcatcaaaccaactactccagcaacatggtagcagtatctcaaggtgtcttcaaaggtcacgtaacgagtttgagcaacatccattgcgaaaccatccaagtgatcaagtgccattcttggcgtaataccgtgtgttagtgcaacttcttggaatgcagcaaaagcaggatcttgcatctcagcaccttcaaaagctgcaagtgtaagcgttctcaatctagccaatctctgagtagcctcttcttctgcagcagcttcagatgcgaaaccatgtgtctggtcatctataacgtcatcacagtgtctacaccaagtgtacagcataagcactgatcttctagtagctgggtcaaacagtttagctgctgtagcaaaggacttggaaccattagccatcgtttgagtagcatgatccaacaaaggtggttgactcatTGTTTTATATTTGTTGTAAAAAGTAGATAATTACTTCCTTGATGATCTGTAAAAAAGAGAAAAAGAAAGCATCTAAGAACTTGAAAAACTACGAATTAGAAAAGACCAAATATGTATTTCTTGCATTGACCAATTTATGCAAGTTTATATATATGTAAATGTAAGTTTCACGAGGTTCTACTAAACTAAACCACCCCCTTGGTTAGAAGAAAAGAGTGTGTGAGAACAGGCTGTTGTTGTCACACGATTCGGACAATTCTGTTTGAAAGAGAGAGAGTAACAGTACGATCGAACGAACTTTGCTCTGGAGATCACAGTGGGCATCATAGCATGTGGTACTAAACCCTTTCCCGCCATTCCAGAACCTTCGATTGCTTGTTACAAAACCTGTGAGCCGTCGCTAGGACCTTGTTGTGTGACGAAATTGGAAGCTGCAATCAATAGGAAGACAGGAAGTCGAGCGTGTCTGGGTTTTTTCAGTTTTGTTCTTTTTGCAAACAAATCACGAGCGACGGTAATTTCTTTCTCGATAAGAGGCCACGTGCTTTATGAGGGTAACATCAATTCAAGAAGGAGGGAAACACTTCCTTTTTCTGGCCCTGATAATAGTATGAGGGTGAAGCCAAAATAAAGGATTCGCGCCCAAATCGGCATCTTTAAATGCAGGTATGCGATAGTTCCTCACTCTTTCCTTACTCACGAGTAATTCTTGCAAATGCCTATTATGCAGATGTTATAATATCTGTGCGTCTTGAGTTGAAGTCAGGAATCTAAAATAAAAATTAAGGTTAATAAAAAGAGGAAAGAAAAAAAAATTAATCGATTTACAGAAACTTGCACACTAAAAATACACAACTAAAAGCAATTACAGTATGGGAAGTCATCGACGTTATCTCTACTATAGTATATTATCATTTCTATTATTATCCTGCTCAGTGGTACTTGCAAAACAAGATAAGACCCCATTCTTTGAAGGTACTTCTTCGAAAAATTCGCGTCTCCCCAAACGGGTAGTCCATCGTTGTAGGATACTGTATATACACCCAGTAGAGTAGGTGACTACTGTATGAGCATACAGTAGTGACAACCACTTACTGTATATAAATACAGTAGTGGTCATCGATACTGTATATAAAACCAGTAGGCAATCGTTTGTACTGTATGTACATACAGTATACCTCGCAACTACTGTATATAAACACAGTAATTAACTTGTAATATTCTAATCAATTGACAATATTATTTAAGGACCTATTGTTTTTTCCAATAGGTGGTTAGCAATCGTCTTACTTTCTAACTTTTCTTACCTTTTACATTTCAGCAATATATATATATATATTTCAAGGATATACCATTCTAAGATCTAAAAAAatgaagaaaaccgtagtgattggtgcaggttttggtggtttagctttggctatacgtctacaagctgcaggtattcctacagtgctattggagcaaagagacaaaccaggaggaagagcttatgtttggcacgatcaaggctttacctttgatgctggtcctacagtcatcactgatcctactgcattggaagctttgttcaccttagctggtagaagaatggaagattatgtccgtctattgcctgtcaagccgttttacagattgtgttgggaatctggtaaaaccctagattacgccaatgacagtgctgaactagaagctcagattacgcagtttaatcccagagatgtcgaaggttacaggagattccttgcctattcccaagctgttttccaagagggttatcttcgtttgggttcagttccattcctgtcctttagggatatgcttagagcaggtcctcagttgttgaagctacaagcatggcaaagtgtgtatcagtctgtttcgagatttatcgaggatgaacatctgagacaagcattctcattccacagtcttctagttggaggtaatccctttaccacatcgagcatatatacgttgattcacgctttggaaagagaatggggagtttggtttcctgaaggtggaacaggtgctttggttaatggtatggtgaagctattcacggatttgggtggagaaatagagctgaatgcaagagtggaagaacttgttgtagcagacaacagagtctcacaagttagacttgctgatggtaggatcttcgatacagatgctgtagcttcaaacgcagatgtagtgaacacttataaaaagttgttgggacatcatcctgttggacaaaagagagcagctgctttggagaggaaatctatgagcaactcgttgtttgtcctttactttgggctgaatcaaccacactcacaactagctcatcacacaatctgctttggtcctagatacagagagctgatagatgaaattttcactggatctgctttagcagacgatttttccctgtacttgcattcaccatgtgttactgatccctctttagcaccacctggttgtgctagcttctatgtactagcacctgtaccacatttgggtaatgctccattagattgggcacaagaaggaccgaaattgagggataggatcttcgactatttggaagaacgttacatgccaggtttgagatctcagttggttacacagaggatattcacaccagctgattttcatgatactctagatgcgcatttaggtagcgctttttccattgagccacttttgacgcaaagtgcttggtttagaccacacaacagagattctgacattgccaatctgtacctagtaggtgcaggaactcatccaggagctggtattcctggagttgtagcttctgctaaagctactgctagtctgatgatcgaggatttgcagtaaTCTCTGCTTTTGTGCGCGTATGTTTATGTATGTACCTCTCTCTCTATTTCTATTTTTAAACCACCCTCTCAATAAAATAAAAATAATAAAGTATTTTTAAGGAAAAGACGTGTTTAAGCACTGACTTTATCTACTTTTTGTACGTTTTCATTGATATAATGTGTTTTGTCTCTCCCTTTTCTACGAAAATTTCAAAAATTGACCAAAAAAAGGAATATATATACGAAAAACTATTATATTTATATATCATAGTGT**TTTT**CGAGACC |
| pTDH3-  FAD1-tPGK1, LexO(6x)-pLEU2m-CrtI-tACT1- [pCCW12-CaBAR1-tENO2, rev] | GGTCTCA**CAGT**AGTTTATCATTATCAATACTCGCCATTTCAAAGAATACGTAAATAATTAATAGTAGTGATTTTCCTAACTTTATTTAGTCAAAAAATTAGCCTTTTAATTCTGCTGTAACCCGTACATGCCCAAAATAGGGGGCGGGTTACACAGAATATATAACATCGTAGGTGTCTGGGTGAACAGTTTATTCCTGGCATCCACTAAATATAATGGAGCCCGCTTTTTAAGCTGGCATCCAGAAAAAAAAAGAATCCCAGCACCAAAATATTGTTTTCTTCACCAACCATCAGTTCATAGGTCCATTCTCTTAGCGCAACTACAGAGAACAGGGGCACAAACAGGCAAAAAACGGGCACAACCTCAATGGAGTGATGCAACCTGCCTGGAGTAAATGATGACACAAGGCAATTGACCCACGCATGTATCTATCTCATTTTCTTACACCTTCTATTACCTTCTGCTCTCTCTGATTTGGAAAAAGCTGAAAAAAAAGGTTGAAACCAGTTCCCTGAAATTATTCCCCTACTTGACTAATAAGTATATAAAGACGGTAGGTATTGATTGTAATTCTGTAAATCTATTTCTTAAACTTCTTAAATTCTACTTTTATAGTTAGTCTTTTTTTTAGTTTTAAAACACCAAGAACTTAGTTTCGAAGGATTCTAGAACTAGTAACatgcagttgagcaaggctgctgagatgtgttatgagataacaaactcttacttacacatagaccagaaatctcagataatagcaagtacacaagaagcgatacggttgacaagaaaatacttactaagtgaaatttttgtacgttggagtccactgaatggggaaatatcattctcgtacaacggaggaaaagattgccaggtattactactgttatatctgagttgcttatgggaatatttcttcattaaggctcaaaattcccaattcgatttcgagtttcaaagcttccccatgcaaagacttccaactgttttcattgatcaagaagaaactttccctacattagagaattttgtactggaaacctcagagcgatattgcctttccttatacgaatcacaaaggcaatctggtgcatcggtcaatatggcagacgcatttagagattttataaagatataccctgaAaccgaagctatagtgataggtattagacacacagacccatttggtgaagcattaaagcctattcaaagaacagattctaactggcctgattttatgaggttgcaacctctcttacactgggacttaaccaatatatggagtttcttactgtattctaatgagccaatttgtggactatatggtaaaggtttcacatcaatcggcggaattaacaactcattgcctaacccacacttgagaaaggactccaataatccagccttgcattttgaatgggaaatcattcatgcatttggcaaggacgcagaaggcgaacgtagttccgctataaacacgtcacctatttccgtggtggataaggaaagattcagcaaataccatgacaattactatcctggctggtatttggttgatgacactttagagagagcaggcaggatcaagaattaaATTGAATTGAATTGAAATCGATAGATCAATTTTTTTCTTTTCTCTTTCCCCATCCTTTACGCTAAAATAATAGTTTATTTTATTTTTTGAATATTTTTTATTTATATACGTATATATAGACTATTATTTATCTTTTAATGATTATTAAGATTTTTATTAAAAAAAAATTCGCTCCTCTTTTAATGCCTTTATGCAGTTTTTTTTTCCCATTCGATATTTCTATGTTCGGGTTCAGCGTATTTTAAGTTTAATAACTCGAAAATTCTGCGTTCGTTAAAGCTTTCGAGAAGGATATTATTTCGAAATAAACCGTGTTGTGTAAGCTTGAAGCCTTTTTGCGCTGCCAATATTCTTATCCATCTATTGTACTCTTTAGATCCAGTATAGTGTATTCTTCCTGCCCCAAACGGGTAGTCCATCGTTGTAGGATACTGTATATACACCCAGTAGAGTAGGTGACTACTGTATGAGCATACAGTAGTGACAACCACTTACTGTATATAAATACAGTAGTGGTCATCGATACTGTATATAAAACCAGTAGGCAATCGTTTGTACTGTATGTACATACAGTATACCTCGCAACTACTGTATATAAACACAGTAATTAACTTGTAATATTCTAATCAATTGACAATATTATTTAAGGACCTATTGTTTTTTCCAATAGGTGGTTAGCAATCGTCTTACTTTCTAACTTTTCTTACCTTTTACATTTCAGCAATATATATATATATATTTCAAGGATATACCATTCTAAGATCTGGAAatgaagaaaaccgtagtgattggtgcaggttttggtggtttagctttggctatacgtctacaagctgcaggtattcctacagtgctattggagcaaagagacaaaccaggaggaagagcttatgtttggcacgatcaaggctttacctttgatgctggtcctacagtcatcactgatcctactgcattggaagctttgttcaccttagctggtagaagaatggaagattatgtccgtctattgcctgtcaagccgttttacagattgtgttgggaatctggtaaaaccctagattacgccaatgacagtgctgaactagaagctcagattacgcagtttaatcccagagatgtcgaaggttacaggagattccttgcctattcccaagctgttttccaagagggttatcttcgtttgggttcagttccattcctgtcctttagggatatgcttagagcaggtcctcagttgttgaagctacaagcatggcaaagtgtgtatcagtctgtttcgagatttatcgaggatgaacatctgagacaagcattctcattccacagtcttctagttggaggtaatccctttaccacatcgagcatatatacgttgattcacgctttggaaagagaatggggagtttggtttcctgaaggtggaacaggtgctttggttaatggtatggtgaagctattcacggatttgggtggagaaatagagctgaatgcaagagtggaagaacttgttgtagcagacaacagagtctcacaagttagacttgctgatggtaggatcttcgatacagatgctgtagcttcaaacgcagatgtagtgaacacttataaaaagttgttgggacatcatcctgttggacaaaagagagcagctgctttggagaggaaatctatgagcaactcgttgtttgtcctttactttgggctgaatcaaccacactcacaactagctcatcacacaatctgctttggtcctagatacagagagctgatagatgaaattttcactggatctgctttagcagacgatttttccctgtacttgcattcaccatgtgttactgatccctctttagcaccacctggttgtgctagcttctatgtactagcacctgtaccacatttgggtaatgctccattagattgggcacaagaaggaccgaaattgagggataggatcttcgactatttggaagaacgttacatgccaggtttgagatctcagttggttacacagaggatattcacaccagctgattttcatgatactctagatgcgcatttaggtagcgctttttccattgagccacttttgacgcaaagtgcttggtttagaccacacaacagagattctgacattgccaatctgtacctagtaggtgcaggaactcatccaggagctggtattcctggagttgtagcttctgctaaagctactgctagtctgatgatcgaggatttgcagtaaTCTCTGCTTTTGTGCGCGTATGTTTATGTATGTACCTCTCTCTCTATTTCTATTTTTAAACCACCCTCTCAATAAAATAAAAATAATAAAGTATTTTTAAGGAAAAGACGTGTTTAAGCACTGACTTTATCTACTTTTTGTACGTTTTCATTGATATAATGTGTTTTGTCTCTCCCTTTTCTACGAAAATTTCAAAAATTGACCAAAAAAAGGAATATATATACGAAAAACTATTATATTTATATATCATAGTGT[ATTTTTCAAACTGCAAATTCAAGAAAAAGCCACGCGTGTGCACCTTTTTTTTCCCCTTCCAGTGCATTATGCAATAGACAGCACGAGTCTTTGAAAAAGTAACTTATAAAACTGTATCAATTTTTAAACCTAAATAGATTCATAAACTATTCGTTAATATAAAGTGTTCTAAACTATGATGAAAAAATAAGCAGAAAAGACTAATAATTCTTAGTTAAAAGCACTCTCGAGTTAACTAGTttaacaatactgagggttgcttgtgacggaagtaatcttatcatcacttgtgctgccgctcgttaggttgctagaggtaccctcactgatagaaaaggatgaataggtgctttggacctctgtcggtgcaacggttaccgtggtttctgattccgcgatatatataacgctataagtgtcctcaacatcgggcacgtctactgtcggtactggtagctggtaaccattctccacagcgacgacgttcgcctctccgttagaattcttatatttaccgaaatatatttgtgcgttgtcgtagtcaaatatagcatagtggtacttaaaaatcaaatttggcagcagaccttcataggcgttatagcctatatcgcatgaagctacatttaaggctacgaatgtagttgagttcctttcaatggtatcgccgactatgtcgaccagctggaagccaatgtcgtacccttgtactgaaaatgttatatttaacccctctatgtacttgatgtcgaaatatatattgccattgtcatcctggatgtagtcgtcgcctaaaattttaaggatgttattaaggaccggagttggggggacgaaaccgttagtgccactatcgatttggtaaagcgtcttctggttgctaacgatggtgtctcccaacttgatctgatttactgtgaacgttggcatcaaccagtagtcaagctggtttatgaaggatatcactttgataaagggcgctcttactaaaggcccgtctatgatgttcgtaattatccctccgaagataatggacgggttctgatcctgaccattgaatgagaatagaacacttttcgtataaccctggtctttaagtagataggggaagttactgtaattcgttttaaaaagttcttgacgagtaaatccaatgcctagtataccggaaaaactgtccatatcagaaatattggccagtcccatagatagattagacagtttccagtcgttactcctaatagtttcattgatgaagtataccgggaactgtcccccgtatccatatttcataattgcggtctggttggagttatagaaacttgatgacgcagagaagttgtatatggagtcaactatcataagatcgctacttcctgaatcgactataacgttagatatgaactggggcggcgtccctaacgctaaattctctatgtaatacatgtgctcatcggatatcgtaaggtctaatttcaccgcaccgccggtattggatacggccacggctagggatgggacaagaagtgaaagaatagtaaataacatACTAGTGATCTTTATTGATATAGTGTTTAAGCGAATGACAGAAGATTAATTTCTTGGTATGTTAGGAAAGAATAAAGGAGAATAAGAATAATTAGAACAATGTAGGATGGAAAGAAAGATTATCAAGCATGCCGACTTTATATACTTGAACGGAGGCAAAGGATGCAAAATTTTCTCACATTTCTTTCTGCCGTTATGTTGGAAGTAAGACTCCCATTATCGCAATACTGCAACACGAATATGCAAAATTTGCTGAGTTATCGCAGATAGTTGTTGCAAAGATAGCGGCGTAGGTGGCCGCGAAATGGGAAATTCCAAAACAAACGGTTTTTTTACTCCTGAGAAATACTTGTACGGGATAATCCAGGGCCTACCACCCACGCTTCGAGGATTGGCTTTTATTTTTTTTTTTTTGGTGGCGTTTTATTTCTTTCCCGCTTTCTGGGACTTGTGCGGAGTTTTGAGAGGGGCGCGCGGCAAAGGATTCCCAAAACGGAAATCAGACGCCAATAGCCAGCACTCAAAGCAGTTCTGGACCCATTCCGATTTTCCCATTTGGTTCTTGCGCGTGCTGATTCCGACACGCGCGTCTATAAATAGCATGAAGTATCCGCACACCGCAGCGTTAGTGAGGTGAGGGTGGCAGCAAGCTAATTCCCGCATCTGGAATCTGAACTGCCCCTTTTGGACTAACCGTGTGGTTCATGGGTG]**TTTT**CGAGACC |
| Readout ORFs | |
| ymTurquoise2 | atggttagtaaaggtgaagaattgttcactggtgtagtgccaattttagtagaattagatggtgacgttaacggtcataagttctctgtaagcggtgaaggtgaaggtgacgcaacatacggtaaattaactttgaaattcatttgtactacaggtaaattaccagttccatggccaactttggttactactttgtcttggggtgttcaatgtttcgctagatacccagatcatatgaagcaacatgatttctttaagtctgctatgccagaaggttacgttcaagaaagaactatcttcttcaaggatgatggtaactataagactagagctgaagttaagttcgaaggagatactttagttaatagaattgaattgaagggtattgattttaaggaagatggtaatattttgggtcataagttggaatataattacttctctgataacgtttacattactgctgataagcaaaagaacggtattaaggcgaacttcaagattagacataacattgaagatggtggtgttcaattagctgatcattaccaacaaaacactccaattggtgacggtccagttttgttgccagataaccattacttgtctactcaatctaagttgtctaaggatccaaacgaaaagagagatcatatggttttgttggaattcgttactgctgctgggatcactttaggtatggatgaattgtataaatag |
| CrtI | atgaagaaaaccgtagtgattggtgcaggttttggtggtttagctttggctatacgtctacaagctgcaggtattcctacagtgctattggagcaaagagacaaaccaggaggaagagcttatgtttggcacgatcaaggctttacctttgatgctggtcctacagtcatcactgatcctactgcattggaagctttgttcaccttagctggtagaagaatggaagattatgtccgtctattgcctgtcaagccgttttacagattgtgttgggaatctggtaaaaccctagattacgccaatgacagtgctgaactagaagctcagattacgcagtttaatcccagagatgtcgaaggttacaggagattccttgcctattcccaagctgttttccaagagggttatcttcgtttgggttcagttccattcctgtcctttagggatatgcttagagcaggtcctcagttgttgaagctacaagcatggcaaagtgtgtatcagtctgtttcgagatttatcgaggatgaacatctgagacaagcattctcattccacagtcttctagttggaggtaatccctttaccacatcgagcatatatacgttgattcacgctttggaaagagaatggggagtttggtttcctgaaggtggaacaggtgctttggttaatggtatggtgaagctattcacggatttgggtggagaaatagagctgaatgcaagagtggaagaacttgttgtagcagacaacagagtctcacaagttagacttgctgatggtaggatcttcgatacagatgctgtagcttcaaacgcagatgtagtgaacacttataaaaagttgttgggacatcatcctgttggacaaaagagagcagctgctttggagaggaaatctatgagcaactcgttgtttgtcctttactttgggctgaatcaaccacactcacaactagctcatcacacaatctgctttggtcctagatacagagagctgatagatgaaattttcactggatctgctttagcagacgatttttccctgtacttgcattcaccatgtgttactgatccctctttagcaccacctggttgtgctagcttctatgtactagcacctgtaccacatttgggtaatgctccattagattgggcacaagaaggaccgaaattgagggataggatcttcgactatttggaagaacgttacatgccaggtttgagatctcagttggttacacagaggatattcacaccagctgattttcatgatactctagatgcgcatttaggtagcgctttttccattgagccacttttgacgcaaagtgcttggtttagaccacacaacagagattctgacattgccaatctgtacctagtaggtgcaggaactcatccaggagctggtattcctggagttgtagcttctgctaaagctactgctagtctgatgatcgaggatttgcagtaa |

**Table S4.** gRNA sequences used for genome engineering.

| **Target gene or locus** | **Sequence 5’→3’** |
| --- | --- |
| STE2 gene in yWS890 | TCACCTCTACTGATGAACTG |
| URA3 gene in yWS890 | GAAGTAACAAAGGAACCTAG |
| LEU2 gene in yWS890 | TTTTTGAACACACATGAACA |
| ARS208a locus | GTCCGCTAAACAAAAGATCT |
| HO locus | AGAATTGATTGCTGCTTATG |
| LEU2 locus | TAACATTAATATTGACAAGG |

**Table S5.** Fungal pheromone proteases.

| **Species  (protease, accession number)** | **Amino acid sequence** |
| --- | --- |
| ***Saccharomyces cerevisiae*** [2]  **(ScBar1, NP_012249)** | MSAINHLCLKLILASFAIINTITALTNDGTGHLEFLLQHEEEMYYATTLDIGTPSQSLTVLFDTGSADFWVMDSSNPFCLPNSNTSSYSNATYNGEEVKPSIDCRSMSTYNEHRSSTYQYLENGRFYITYADGTFADGSWGTETVSINGIDIPNIQFGVAKYATTPVSGVLGIGFPRRESVKGYEGAPNEYYPNFPQILKSEKIIDVVAYSLFLNSPDSGTGSIVFGAIDESKFSGDLFTFPMVNEYPTIVDAPATLAMTIQGLGAQNKSSCEHETFTTTKYPVLLDSGTSLLNAPKVIADKMASFVNASYSEEEGIYILDCPVSVGDVEYNFDFGDLQISVPLSSLILSPETEGSYCGFAVQPTNDSMVLGDVFLSSAYVVFDLDNYKISLAQANWNASEVSKKLVNIQTDGSISGAKIATAEPWSTNEPFTVTSDIYSSTGCKSRPFLQSSTASSLIAETNVQSRNCSTKMPGTRSTTVLSKPTQNSAMHQSTGAVTQTSNETKLELSSTMANSGSVSLPTSNSIDKEFEHSKSQTTSDPSVAEHSTFNQTFVHETKYRPTHKTVITETVTKYSTVLINVCKPTY |
| ***Candida albicans***  [3]  **(CaBar1, KGQ96729)** | MLFTILSLLVPSLAVAVSNTGGAVKLDLTISDEHMYYIENLALGTPPQFISNVIVDSGSSDLMIVDSIYNFSASSSFYNSNQTAIMKYGYGGQFPVYFINETIRSNDWKLSNLSMGLANISDMDSFSGILGIGFTRQELFKTNYSNFPYLLKDQGYTKSVLFSFNGQDQNPSIIFGGIITNIIDGPLVRAPFIKVISFINQLDYWLMPTFTVNQIKLGDTIVSNQKTLYQIDSGTNGFVPPTPVLNNILKILGDDYIQDDNGNIYFDIKYIEGLNITFSVQGYDIGFQLVDIVGDTIERNSTTFVALNVASCDIGYNAYEGLLPNLIFKYHYAIFDYDNAQIYFGKYKNSNGEANVVAVENGYQLPVPTVDVPDVEDTYSVIYIAESETTVTVAPTEVQSTYSSFSISEGTSSNLTSGSTSDDKITSVTSNPQYC |
| ***Schizosaccharomyces pombe*** [4]  **(SpSxa2, NP_593043)** | MLSLFLKSLFAIIIIELTIIHALPTYTVHWKCSIQQANTSSASSNQTVQPRQHAAPSSDRIKSLPEFKGSLPELYSGYLEANSDKSLFYTYAPAVVDSETFIVWLQGGPGCAGTLGFFSENGPIEISQSSPSPSLNPESWTNFANMLWLDQPFGTGYSQGQAAYTTTIEEASSDFVNALKSFYQKFPHLMKKKLYLVGESYGSIWSANFAEALLSEPSLNINFMGVGIVSGLTADYETQEQITASIWVEHISKLGYYFNNTSSTISEEFKKRNKECQYDSVLNRLTFPTEQYPIWRPEYNFSTSTSLRKREALDGEDIGNVFNSISGCDLYSLSNFLLYLENSCVITYDVSLDCSFNEYNDPLITYLNREDVRSSLHATKASTALTSGEGVFADGCNFDLYKKIVSNNVESVLVEIIPRLTEKYKVSFLAGALDLQILWTGTLLALQNTTWNGWQGFTQSPGSLETTNGFTLDERNLAFTLSNSVGHMAPSKDPQMVREWLENTLLY |
| ***Schizosaccharomyces octosporus***  **(SoSxa2, XP_013015964)** | MNLAVHVLLLSFLLYTVHSFPAYPPYPIYPRSSLNSSNASSVPIPQPSYNSSSKAMNPDLVTSLPEYSGSLPELYSGYLSAENFKQIFYTFAPAITPTDKFIVWLQGGPGCAGTLGFFSENGPIILDPNHQTPQDNPNSWTRFANMMWIDQPFGTGYSQGSPLASTVSIATSDFIQTLKSFYAKFPEMKEKELYLVGESYGSVWGAYFAQALRSDPELHAIPFGGLGIISGLLSDYETQNTITASMWLDHVNKMGLYYTNESAEVSTFFAAKNQQCGYAEVLERMQFPAEQTPIPKPRVNLTNSFSKKKRQADSDSDSGNIKEAFQMITDCDTYTLTDFLLYLKNHCMITYDITLDCSFNSNNDPLTTYLNRKDVQKQLHAIHVESALTSSKGIFGNGCNYDVYGDIVKNGAPSILEEAIPELVQNYKVSFLAGALDFQLLWTGSLLALQNTTWNGWQGFQEIPDLNSPFGFTLDERNLAFTLSNKVGHMAPSKDPSMVIDWLQKTLLG |
| ***Schizosaccharomyces japonicus***  **(SjSxa2, XP_002172242)** | MVVLHRLLLSLWPAGAMLLVSSFLLNLPTTSAIPIYSDPHQCSLRVSVRTDVEGEQQQTQAMGSVVIASNVTSSDAPTENPTSSAPTTFSHTVNSTFPTHTPTTSTSTNTPTDARVSSLPLLNGDMPALYAGYLNVSASSTSPHSIFYAYAPSEQGSDDLIVWLQGGPGCAGTIGLFAENGPVRFGPGDSAPVRSEHSWTRYADVLYLDQPFGTGFSYAVTEADYTTTMDAASAEFIAFLDGFFDAYPDTASKRLFFVGESYGSFWGAYFARALADRPDIYDRIVSVGIVSGLSTTHDTQYSVSNSLWLSHLNKLGYYTQDKSTWNSFSRVNARCGYDKVMQEFTFPASQTPLSSISTSTSSSSTSSGAGAGATVSSLTDSSGNASYTNSSSSAEVDVFSYFPSCDTNDATYMLMYLQNTCLISYDVSIDCTTASQLTDHLTPYLNRADVREALHVPSSVAAFSTANGVFGDGCNYDLLQKINTVTYEPVLKSVVPSLIADGKRVAFLSGALDYQIPWSGTLLAMQNTTWAGWQGFQTEPDWDNPVGFTLNERGLSFTLLNSVGHMASMNDPDRVNRWLQEQVL |

**Table S6.** Fungal pheromone GPCRs.

| **Species  (GPCR, accession number)** | **Amino acid sequence** |
| --- | --- |
| ***Saccharomyces cerevisiae*** [5]  **(ScSte2, D6VTK4)** | MSDAAPSLSNLFYDPTYNPGQSTINYTSIYGNGSTITFDELQGLVNSTVTQAIMFGVRCGAAALTLIVMWMTSRSRKTPIFIINQVSLFLIILHSALYFKYLLSNYSSVTYALTGFPQFISRGDVHVYGATNIIQVLLVASIETSLVFQIKVIFTGDNFKRIGLMLTSISFTLGIATVTMYFVSAVKGMIVTYNDVSATQDKYFNASTILLASSINFMSFVLVVKLILAIRSRRFLGLKQFDSFHILLIMSCQSLLVPSIIFILAYSLKPNQGTDVLTTVATLLAVLSLPLSSMWATAANNASKTNTITSDFTTSTDRFYPGTLSSFQTDSINNDAKSSLRSRLYDLYPRRKETTSDKHSERTFVSETADDIEKNQFYQLPTPTSSKNTRIGPFADASYKEGEVEPVDMYTPDTAADEEARKFWTEDNNNL |
| ***Candida albicans***  [6]  **(CaSte2)** | MNINSTFIPDKPGDIIISYSIPGLDQPIQIPFHSLDSFQTDQAKIALVMGITIGSCSMTLIFLISIMYKTNKLTNLKLKLKLKYILQWINQKIFTKKRNDNKQQQQQQQQQIESSSYNNTTTTLGGYKLFLFYLNSLILLIGIIRSGCYLNYNLGPLNSLSFVFTGWYDGSSFISSDVTNGFKCILYALVEISLGFQVYVMFKTSNLKIWGIMASLLSIGLGLIVVAFQINLTILSHIRFSRAISTNRSEEESSSSLSSDSVGYVINSIWMDLPTILFSISINIMTILLIGKLIIAIRTRRYLGLKQFDSFHILLIGFSQTLIIPSIILVVHYFYLSQNKDSLLQQISLLLIILMLPLSSLWAQTANNTHNINSSPSLSFISRHHLSDSSRSGGSNTIVSNGGSNGGGGGGGNFPVSGIDAQLPPDIEKILHEDNNYKLLNSNNESVNDGDIIINDEGMITKQITIKRV |
| ***Schizosaccharomyces pombe*** [6]  **(SpSte2, NP_594722)** | MRQPWWKDFTIPDASAIIHQNITIVSIVGEIEVPVSTIDAYERDRLLTGMTLSAQLALGVLTILMVCLLSSSEKRKHPVFVFNSASIVAMCLRAILNIVTICSNSYSILVNYGFILNMVHMYVHVFNILILLLAPVIIFTAEMSMMIQVRIICAHDRKTQRIMTVISACLTVLVLAFWITNMCQQIQYLLWLTPLSSKTIVGYSWPYFIAKILFAFSIIFHSGVFSYKLFRAILIRKKIGQFPFGPMQCILVISCQCLIVPATFTIIDSFIHTYDGFSSMTQCLLIISLPLSSLWASSTALKLQSMKTSSAQGETTEVSIRVDRTFDIKHTPSDDYSISDESETKKWT |
| ***Schizosaccharomyces octosporus***  [6]  **(SoSte2, XP_013018801)** | MREPWWKNYYTMNGTQVQNQSIPILSTQGYIQVPLSTIDKAERNRILTGMTVSAQLALGVLIMVMSILLSSPEKRKTPVFIVNSASIISMCIRAILMIVNLCSESYSLAVMYGFVFELVGQYVHVFDILVMIIGTIIIITAEVSMLLQVRIICAHDRKTQRIVTCISSGLSLIVVAFWFTDMCQEIKYLLWLTPYNNHQISGYYWVYFVGKILFAVSIMFHSAVFSYKLFHAIQIRKKIGQFPFGPMQCILIISCQCLFVPAIFTIIDSFIHTYDGFSSMTQCLLIVSLPLSSLWASSTALKLQSLKSTTSPGDTTQVSIRVDRTYDIKRIPTEELSSVDETEIKKWP |
| ***Schizosaccharomyces japonicus***  [6]  **(SjSte2, XP_002173203)** | MYSWDEFRSPKQAEVLNQTVTLETIVSTIQLPISEIDSMERNRLLTGMTVAVQVGLGSFILVLMCIFSSSEKRKKPVFIFNFAGNLVMTLRAIFEVIVLASNNYSIAVQYGFAFAAVRQYVHAFNIIILLLGPFILFIAEMSLMLQVRIICSQHRPTMITTTVISCIFTVVTLAFWITDMSQEIAYQLFLKNYNMKQIVGYSWLYFIAKITFAASIIFHSSVFSFKLMRAIYIRRKIGQFPFGPMQCIFIVSCQCLIVPAIFTLIDSFTHTYDGFSSMTQCLLIISLPLSSLWATHTAQKLQTMKDNTNPPSGTQLTIRVDRTFDMKFVSDSSDGSFTEKTEETLP |

**Table S7.** Yeast codon-optimized sequences without internal BsaI and NotI restriction sites.

| **Sequence name** | **Nucleotide sequence** |
| --- | --- |
| **ScSte2** | ATGTCTGATGCGGCTCCTTCATTGAGCAATCTATTTTATGATCCAACGTATAATCCTGGTCAAAGCACCATTAACTACACTTCCATATATGGGAATGGATCTACCATCACTTTCGATGAGTTGCAAGGTTTAGTTAACAGTACTGTTACTCAGGCCATTATGTTTGGTGTCAGATGTGGTGCAGCTGCTTTGACTTTGATTGTCATGTGGATGACATCGAGAAGCAGAAAAACGCCGATTTTCATTATCAACCAAGTTTCATTGTTTTTAATCATTTTGCATTCTGCACTCTATTTTAAATATTTACTGTCTAATTACTCTTCAGTGACTTACGCTCTCACCGGATTTCCTCAGTTCATCAGTAGAGGTGACGTTCATGTTTATGGTGCTACAAATATAATTCAAGTCCTTCTTGTGGCTTCTATTGAGACTTCACTGGTGTTTCAGATAAAAGTTATTTTCACAGGCGACAACTTCAAAAGGATAGGTTTGATGCTGACGTCGATATCTTTCACTTTAGGGATTGCTACAGTTACCATGTATTTTGTAAGCGCTGTTAAAGGTATGATTGTGACTTATAATGATGTTAGTGCCACCCAAGATAAATACTTCAATGCATCCACAATTTTACTTGCATCCTCAATAAACTTTATGTCATTTGTCCTGGTAGTTAAATTGATTTTAGCTATTAGATCAAGAAGATTCCTTGGTCTCAAGCAGTTCGATAGTTTCCATATTTTACTCATAATGTCATGTCAATCTTTGTTGGTTCCATCGATAATATTCATCCTCGCATACAGTTTGAAACCAAACCAGGGAACAGATGTCTTGACTACTGTTGCAACATTACTTGCTGTATTGTCTTTACCATTATCATCAATGTGGGCCACGGCTGCTAATAATGCATCCAAAACAAACACAATTACTTCAGACTTTACAACATCCACAGATAGGTTTTATCCAGGCACGCTGTCTAGCTTTCAAACTGATAGTATCAACAACGATGCTAAAAGCAGTCTCAGAAGTAGATTATATGACCTATATCCTAGAAGGAAGGAAACAACATCGGATAAACATTCGGAAAGAACTTTTGTTTCTGAGACTGCAGATGATATAGAGAAAAATCAGTTTTATCAGTTGCCCACACCTACGAGTTCAAAAAATACTAGGATAGGACCGTTTGCTGATGCAAGTTACAAAGAGGGAGAAGTTGAACCCGTCGACATGTACACTCCCGATACGGCAGCTGATGAGGAAGCCAGAAAGTTCTGGACTGAAGATAATAATAATTTATAG |
| **CaSte2** | ATGAATATCAATTCAACTTTCATACCTGATAAACCAGGCGATATAATTATTAGTTATTCAATTCCAGGATTAGATCAACCAATTCAAATTCCTTTCCATTCATTAGATTCATTTCAAACCGATCAAGCTAAAATAGCTTTAGTCATGGGGATAACTATTGGGAGTTGTTCAATGACATTAATTTTTTTGATTTCTATAATGTATAAAACTAATAAATTAACAAATTTAAAATTAAAATTAAAATTAAAATATATCTTGCAATGGATAAATCAAAAAATCTTCACCAAAAAAAGGAATGACAACAAACAACAACAACAACAACAACAACAACAAATTGAATCATCATCATATAACAATACTACTACTACGCTGGGGGGTTATAAATTATTTTTATTTTATCTTAATTCATTGATTTTATTAATTGGTATTATTCGATCAGGTTGTTATTTAAATTATAATTTAGGTCCATTAAATTCACTTAGTTTTGTATTTACTGGTTGGTATGATGGATCATCATTTATATCATCCGATGTAACTAATGGATTTAAATGTATTTTATATGCTTTAGTGGAAATTTCATTAGGTTTCCAAGTTTATGTGATGTTCAAAACTTCAAATTTAAAAATTTGGGGGATAATGGCATCATTATTATCAATTGGTTTAGGATTGATTGTTGTTGCCTTTCAAATCAATTTAACAATTTTATCTCATATTCGATTTTCCCGGGCTATATCAACTAACAGAAGTGAAGAAGAATCATCATCATCATTATCATCTGATTCGGTTGGGTATGTGATTAATTCAATATGGATGGATTTACCAACAATATTATTTTCCATTAGTATTAATATAATGACAATATTATTGATTGGTAAACTTATAATTGCTATTAGAACAAGACGTTATTTAGGATTGAAACAATTTGATAGTTTCCATATTTTATTAATTGGTTTCAGTCAAACATTAATTATTCCTTCAATTATTTTGGTGGTTCATTATTTTTATTTATCACAAAATAAAGATTCTTTATTACAACAAATTAGTCTTTTATTGATTATTTTAATGTTACCATTAAGTTCTTTATGGGCTCAAACTGCTAATAATACTCATAATATTAATTCATCTCCAAGTTTATCATTCATATCTCGTCATCATCTGTCTGATAGTAGTCGTAGTGGTGGTTCCAATACAATTGTTAGTAATGGTGGTAGTAATGGTGGTGGTGGTGGTGGTGGGAATTTCCCTGTTTCAGGTATTGATGCACAATTACCACCTGATATTGAAAAAATCTTACATGAAGATAATAATTATAAATTACTTAATAGTAATAATGAAAGTGTAAATGATGGAGATATTATCATTAATGATGAAGGTATGATTACTAAACAAATCACCATCAAAAGAGTGTAG |
| **SpSte2** | ATGAGACAACCATGGTGGAAAGACTTTACTATTCCCGATGCATCCGCAATTATTCACCAAAATATTACCATTGTCTCTATTGTAGGAGAGATTGAAGTGCCAGTTTCAACAATTGATGCATATGAAAGAGATAGACTTTTAACTGGAATGACTTTGTCTGCCCAACTTGCTTTAGGAGTCCTTACCATTTTGATGGTTTGTCTATTGTCATCATCCGAAAAACGAAAACACCCAGTTTTTGTTTTTAATTCGGCAAGTATTGTTGCAATGTGTCTTCGGGCCATTTTGAATATAGTGACCATATGCAGCAATAGCTACAGTATCCTGGTTAATTACGGGTTTATCTTAAACATGGTTCATATGTATGTCCATGTGTTTAATATTTTAATTTTGTTGCTTGCACCGGTCATCATTTTTACTGCTGAGATGAGCATGATGATTCAAGTTCGTATAATTTGTGCACATGATAGAAAGACACAAAGGATAATGACTGTTATTAGTGCCTGCTTAACTGTTTTGGTTCTCGCATTTTGGATTACTAACATGTGTCAACAGATTCAGTATCTGTTATGGTTAACTCCACTTAGCAGCAAGACCATTGTTGGATACTCTTGGCCCTACTTTATTGCTAAAATACTTTTTGCTTTTAGCATTATTTTTCACAGTGGTGTTTTTTCATACAAACTCTTTCGTGCCATATTAATACGGAAAAAAATTGGGCAATTTCCATTTGGTCCGATGCAGTGTATTTTAGTTATTAGCTGCCAATGTCTTATTGTTCCAGCTACCTTTACTATAATAGATAGTTTTATCCATACGTATGATGGCTTTAGCTCTATGACTCAATGTCTGCTAATCATTTCTCTTCCTCTTTCGAGTTTATGGGCGTCTAGTACAGCTCTGAAATTGCAAAGCATGAAAACTTCATCTGCGCAAGGAGAAACCACCGAGGTTTCGATTAGAGTTGATAGAACGTTTGATATCAAACATACTCCCAGTGACGATTATTCGATTTCTGATGAATCTGAAACTAAAAAGTGGACGTAG |
| **SoSte2** | ATGCGTGAACCATGGTGGAAGAACTACTACACCATGAACGGTACCCAAGTCCAAAACCAATCCATCCCAATTTTGTCCACCCAAGGTTACATTCAAGTTCCATTGTCCACCATCGATAAGGCTGAAAGAAACAGAATTTTGACTGGTATGACCGTTTCTGCTCAATTGGCCTTGGGTGTCTTGATCATGGTCATGTCTATTTTGTTGTCCTCCCCAGAAAAGAGAAAGACCCCAGTTTTCATCGTCAACTCTGCCTCTATCATTTCCATGTGTATTAGAGCTATCTTGATGATTGTCAACTTGTGTTCTGAATCCTACTCTTTGGCTGTTATGTACGGTTTCGTCTTCGAATTGGTTGGTCAATACGTTCACGTTTTTGACATTTTGGTTATGATTATTGGTACCATCATCATTATTACCGCTGAAGTTTCCATGTTGTTGCAAGTCAGAATTATTTGTGCTCACGACAGAAAGACTCAAAGAATTGTTACCTGTATCTCTTCTGGTTTATCCTTGATCGTCGTTGCCTTCTGGTTCACTGATATGTGTCAAGAAATTAAGTACTTGTTGTGGTTGACCCCATACAACAACCACCAAATCTCTGGTTACTACTGGGTTTACTTCGTCGGTAAGATCTTGTTCGCCGTTTCCATTATGTTCCACTCTGCCGTCTTCTCCTACAAGTTGTTCCACGCTATCCAAATTAGAAAGAAGATTGGTCAATTCCCATTCGGTCCAATGCAATGTATTTTAATTATTTCCTGTCAATGTTTGTTCGTTCCAGCTATTTTCACTATCATCGACTCTTTCATCCACACTTACGACGGTTTTTCCTCCATGACCCAATGTTTGTTGATCGTCTCTTTGCCATTGTCCTCCTTGTGGGCCTCTTCCACTGCTTTAAAGTTGCAATCTTTGAAGTCTACCACCTCTCCAGGTGACACTACTCAAGTTTCCATTAGAGTCGACAGAACCTACGACATCAAGAGAATCCCAACTGAAGAATTGTCTTCTGTTGACGAAACCGAAATCAAGAAGTGGCCATAG |
| **SjSte2** | ATGTACTCCTGGGACGAATTCAGATCCCCAAAGCAAGCTGAAGTTTTGAACCAAACCGTTACCTTGGAAACTATTGTTTCCACCATTCAATTGCCAATCTCTGAAATTGACTCCATGGAAAGAAACAGATTGTTGACCGGTATGACTGTCGCTGTTCAAGTTGGTTTAGGTTCCTTCATTTTAGTTTTGATGTGTATTTTCTCTTCCTCTGAAAAGAGAAAGAAGCCAGTCTTCATCTTCAACTTCGCTGGTAACTTGGTTATGACTTTGAGAGCTATTTTCGAAGTTATCGTTTTGGCTTCTAACAACTACTCTATCGCTGTTCAATACGGTTTCGCTTTTGCTGCCGTCAGACAATACGTTCACGCCTTCAACATTATCATCTTGTTGTTGGGTCCATTCATCTTGTTCATCGCTGAAATGTCTTTGATGTTGCAAGTTAGAATCATTTGTTCCCAACACAGACCAACTATGATTACCACCACTGTTATCTCTTGTATTTTCACTGTTGTTACCTTGGCCTTCTGGATCACCGACATGTCTCAAGAAATTGCTTACCAATTGTTCTTGAAAAACTACAACATGAAGCAAATTGTTGGTTACTCCTGGTTGTACTTTATCGCTAAGATCACCTTCGCTGCTTCCATTATCTTCCATTCCTCCGTCTTCTCCTTCAAATTGATGCGTGCTATTTACATTCGTAGAAAGATCGGTCAATTCCCATTCGGTCCAATGCAATGTATCTTCATTGTTTCCTGTCAATGTTTGATCGTTCCAGCTATTTTCACTTTGATCGATTCTTTCACCCACACTTACGATGGTTTCTCCTCCATGACTCAATGTTTGTTGATCATCTCCTTACCATTGTCTTCCTTGTGGGCCACCCACACCGCTCAAAAGTTGCAAACCATGAAGGATAACACTAACCCACCATCTGGTACCCAATTAACCATCAGAGTTGATCGTACTTTCGACATGAAGTTCGTTTCCGACTCCTCTGACGGTTCTTTCACTGAAAAGACCGAAGAAACTTTGCCATAG |
| **ScBar1** | ATGTCTGCAATTAATCATCTTTGTTTGAAACTTATTTTGGCGAGTTTCGCGATTATTAACACCATTACTGCTTTAACAAACGATGGCACTGGTCACTTAGAATTCCTTTTACAACACGAAGAGGAGATGTATTACGCAACAACCTTAGATATAGGTACACCGTCCCAAAGTCTGACAGTGTTGTTTGATACCGGATCTGCCGATTTTTGGGTTATGGATTCTAGCAATCCCTTCTGCTTACCAAATTCAAATACGTCATCCTATTCAAACGCAACTTATAATGGCGAAGAAGTTAAGCCTTCAATTGATTGCAGGTCTATGAGTACTTATAATGAGCATAGATCTTCCACCTACCAATATCTGGAAAATGGTAGGTTTTACATCACATATGCTGACGGAACATTTGCTGACGGTAGTTGGGGGACGGAAACTGTATCAATTAATGGAATTGACATCCCCAATATCCAGTTCGGAGTTGCCAAGTATGCTACGACACCCGTTAGTGGTGTTCTTGGAATTGGGTTTCCTAGAAGAGAGTCCGTTAAGGGCTATGAAGGTGCTCCTAATGAATATTATCCTAATTTTCCTCAGATTTTAAAAAGTGAAAAAATAATCGATGTGGTCGCGTATTCGCTGTTCTTAAACTCACCTGATTCAGGTACTGGTTCGATTGTTTTTGGTGCCATTGATGAATCAAAGTTTTCTGGTGATTTGTTCACTTTCCCTATGGTAAATGAATATCCCACAATAGTCGACGCTCCTGCAACTTTAGCAATGACTATACAAGGATTAGGTGCCCAAAACAAAAGTAGTTGTGAACATGAAACGTTTACGACGACCAAGTATCCAGTTTTGTTGGACTCAGGAACCTCGCTATTGAATGCGCCCAAGGTCATAGCAGATAAAATGGCTTCTTTTGTAAATGCGTCCTATAGTGAAGAGGAAGGTATATATATATTAGACTGTCCAGTATCTGTAGGTGACGTGGAATACAATTTTGATTTCGGCGATTTGCAAATAAGTGTTCCACTGTCTAGTTTGATTTTAAGTCCCGAGACAGAAGGCAGCTATTGTGGGTTTGCGGTCCAGCCAACAAACGATTCGATGGTTCTGGGTGATGTGTTCCTGTCCTCTGCATACGTCGTATTCGATCTCGATAATTATAAGATATCTTTAGCACAGGCAAATTGGAACGCAAGCGAAGTTTCGAAAAAGCTAGTAAATATTCAAACAGATGGGTCTATTTCAGGTGCCAAAATTGCTACAGCTGAACCCTGGTCCACCAATGAACCATTTACAGTCACCTCTGACATTTATTCATCTACAGGCTGCAAGAGTAGGCCTTTTCTTCAATCATCGACAGCCTCTTCGCTTATTGCAGAAACCAACGTACAAAGTCGCAACTGCTCTACGAAGATGCCAGGCACTAGATCAACTACTGTCTTAAGTAAGCCTACTCAAAATAGTGCTATGCATCAAAGTACAGGCGCTGTCACACAAACCTCAAATGAAACTAAATTAGAATTATCCTCGACTATGGCAAATTCGGGCAGTGTCTCGCTTCCCACTTCGAATTCAATAGACAAAGAGTTCGAACATTCGAAATCTCAAACTACCAGCGATCCAAGTGTAGCAGAGCATTCTACGTTTAACCAAACGTTTGTACATGAAACTAAATATCGGCCTACTCATAAGACAGTCATAACAGAAACTGTCACGAAGTATTCTACAGTCTTAATAAATGTCTGTAAACCAACATATTAA |
| **CaBar1** | ATGTTATTTACTATTCTTTCACTTCTTGTCCCATCCCTAGCCGTGGCCGTATCCAATACCGGCGGTGCGGTGAAATTAGACCTTACGATATCCGATGAGCACATGTATTACATAGAGAATTTAGCGTTAGGGACGCCGCCCCAGTTCATATCTAACGTTATAGTCGATTCAGGAAGTAGCGATCTTATGATAGTTGACTCCATATACAACTTCTCTGCGTCATCAAGTTTCTATAACTCCAACCAGACCGCAATTATGAAATATGGATACGGGGGACAGTTCCCGGTATACTTCATCAATGAAACTATTAGGAGTAACGACTGGAAACTGTCTAATCTATCTATGGGACTGGCCAATATTTCTGATATGGACAGTTTTTCCGGTATACTAGGCATTGGATTTACTCGTCAAGAACTTTTTAAAACGAATTACAGTAACTTCCCCTATCTACTTAAAGACCAGGGTTATACGAAAAGTGTTCTATTCTCATTCAATGGTCAGGATCAGAACCCGTCCATTATCTTCGGAGGGATAATTACGAACATCATAGACGGGCCTTTAGTAAGAGCGCCCTTTATCAAAGTGATATCCTTCATAAACCAGCTTGACTACTGGTTGATGCCAACGTTCACAGTAAATCAGATCAAGTTGGGAGACACCATCGTTAGCAACCAGAAGACGCTTTACCAAATCGATAGTGGCACTAACGGTTTCGTCCCCCCAACTCCGGTCCTTAATAACATCCTTAAAATTTTAGGCGACGACTACATCCAGGATGACAATGGCAATATATATTTCGACATCAAGTACATAGAGGGGTTAAATATAACATTTTCAGTACAAGGGTACGACATTGGCTTCCAGCTGGTCGACATAGTCGGCGATACCATTGAAAGGAACTCAACTACATTCGTAGCCTTAAATGTAGCTTCATGCGATATAGGCTATAACGCCTATGAAGGTCTGCTGCCAAATTTGATTTTTAAGTACCACTATGCTATATTTGACTACGACAACGCACAAATATATTTCGGTAAATATAAGAATTCTAACGGAGAGGCGAACGTCGTCGCTGTGGAGAATGGTTACCAGCTACCAGTACCGACAGTAGACGTGCCCGATGTTGAGGACACTTATAGCGTTATATATATCGCGGAATCAGAAACCACGGTAACCGTTGCACCGACAGAGGTCCAAAGCACCTATTCATCCTTTTCTATCAGTGAGGGTACCTCTAGCAACCTAACGAGCGGCAGCACAAGTGATGATAAGATTACTTCCGTCACAAGCAACCCTCAGTATTGTTAA |
| **SpSxa2** | ATGTTAAGTCTTTTCTTAAAGTCTTTGTTCGCAATTATTATCATTGAACTTACTATCATCCACGCCCTTCCGACGTACACGGTGCACTGGAAGTGTAGCATACAACAAGCCAATACTTCCTCAGCATCCTCTAACCAGACCGTCCAGCCGCGTCAGCACGCAGCGCCGAGTAGCGATCGTATAAAGTCCCTACCCGAATTTAAGGGATCACTGCCTGAGTTATACTCAGGTTACTTGGAGGCAAATTCTGACAAGTCACTATTTTACACATATGCGCCTGCAGTTGTAGACTCTGAAACGTTCATCGTGTGGTTGCAGGGCGGACCGGGCTGCGCGGGCACTCTAGGTTTCTTCAGTGAAAACGGCCCGATTGAGATTAGCCAGTCATCTCCTTCTCCTTCATTAAATCCTGAGAGTTGGACCAATTTCGCAAACATGTTGTGGCTGGACCAGCCTTTTGGTACTGGGTATAGCCAGGGGCAGGCGGCTTACACTACCACCATAGAGGAAGCCTCTAGCGACTTCGTCAATGCCCTAAAATCTTTTTACCAAAAATTCCCGCATTTGATGAAAAAGAAGCTGTATCTTGTTGGGGAAAGCTATGGCAGTATATGGAGTGCCAATTTTGCGGAGGCCCTACTTAGCGAACCAAGTCTAAATATCAATTTTATGGGTGTGGGTATTGTCAGCGGACTAACAGCCGATTATGAGACTCAGGAACAAATAACGGCAAGTATATGGGTTGAGCATATATCCAAACTGGGTTACTATTTTAACAATACCTCCTCTACCATATCAGAAGAGTTCAAGAAACGTAACAAAGAGTGTCAGTACGACTCCGTATTGAATAGATTAACGTTTCCCACCGAACAGTATCCCATATGGCGTCCTGAATACAATTTTTCAACCTCAACCAGTCTTAGAAAGAGGGAAGCACTGGATGGGGAAGATATCGGTAACGTTTTCAATTCCATCTCTGGTTGTGATCTGTATTCATTATCTAACTTTCTACTGTACCTTGAAAACAGTTGCGTGATTACATACGACGTCAGTCTTGACTGCTCCTTCAATGAATATAACGATCCATTGATTACATACTTGAACAGAGAAGACGTCAGATCCTCCTTGCACGCAACCAAGGCCTCCACGGCGTTAACATCCGGAGAGGGTGTCTTCGCCGATGGATGTAACTTCGATCTGTATAAGAAGATTGTCTCAAACAATGTCGAATCTGTGCTAGTAGAGATCATACCTCGTTTGACAGAGAAATATAAAGTCAGTTTCTTAGCAGGTGCTCTGGACCTACAAATTCTATGGACGGGGACTTTGTTAGCTCTACAGAACACTACATGGAACGGGTGGCAAGGATTTACACAATCTCCCGGCTCTCTGGAAACGACGAATGGTTTCACGCTGGATGAGAGAAATCTTGCGTTTACCCTAAGTAACAGCGTGGGCCATATGGCTCCAAGCAAGGATCCACAAATGGTGAGAGAATGGCTAGAGAACACCCTTCTTTACTAA |
| **SoSxa2** | ATGAACTTAGCCGTCCATGTACTGCTGTTGTCTTTCTTGTTGTACACTGTTCACAGTTTCCCCGCATATCCGCCATATCCAATATATCCGAGATCATCTCTGAACTCAAGTAACGCCTCCAGCGTACCAATACCACAACCCTCATACAATTCATCTTCCAAGGCCATGAATCCTGACCTGGTCACTAGCTTGCCAGAGTATTCAGGTAGTCTACCAGAATTATACAGCGGATACCTTAGCGCCGAGAACTTCAAGCAGATCTTTTATACTTTTGCACCTGCCATTACCCCGACTGACAAATTCATAGTTTGGCTGCAAGGGGGTCCTGGGTGCGCAGGCACCTTAGGCTTCTTTTCCGAAAACGGTCCAATTATCTTGGACCCCAATCACCAAACTCCACAAGACAATCCCAATAGCTGGACTCGTTTCGCAAACATGATGTGGATTGACCAACCCTTTGGAACAGGGTATTCTCAAGGCTCCCCATTGGCAAGTACAGTTTCAATCGCTACTTCAGATTTTATCCAAACTCTGAAGTCTTTTTACGCAAAATTCCCTGAGATGAAGGAAAAAGAGTTGTATCTTGTGGGCGAGTCCTATGGATCCGTGTGGGGCGCGTATTTTGCCCAAGCCTTGAGGTCCGATCCCGAGCTGCATGCTATACCTTTTGGGGGGCTAGGTATCATAAGCGGCCTTCTATCAGACTATGAAACTCAGAATACTATAACAGCGTCAATGTGGCTTGATCATGTTAACAAAATGGGCTTGTATTATACTAATGAGTCCGCCGAGGTATCTACGTTTTTCGCTGCAAAGAATCAACAATGTGGTTACGCAGAAGTCCTAGAGAGAATGCAATTCCCAGCCGAACAGACTCCGATACCTAAGCCGCGTGTCAATTTGACTAACTCTTTCTCAAAAAAGAAAAGGCAGGCCGACTCTGACTCTGACTCCGGCAATATAAAGGAAGCATTTCAAATGATCACGGACTGTGATACTTATACGTTAACGGACTTTCTTCTTTACCTGAAAAACCATTGTATGATAACATACGATATTACGCTTGATTGCTCATTCAATTCAAACAACGACCCTCTAACAACCTATCTAAATAGAAAAGACGTACAGAAGCAACTGCACGCTATTCATGTCGAGAGCGCTTTAACATCCTCCAAAGGCATCTTTGGTAATGGATGCAACTATGACGTCTATGGTGACATTGTCAAAAATGGCGCACCTTCAATACTGGAAGAAGCGATACCTGAGTTAGTACAGAACTATAAAGTCAGTTTCTTAGCCGGGGCCTTAGACTTTCAATTGCTTTGGACTGGTAGTTTGCTGGCACTACAAAACACGACCTGGAACGGTTGGCAGGGTTTTCAAGAGATCCCTGACCTAAACTCCCCCTTTGGTTTTACGCTTGACGAGCGTAATCTGGCGTTCACGCTTAGCAACAAGGTAGGACATATGGCCCCGAGCAAGGACCCCAGCATGGTAATAGATTGGCTGCAAAAAACCCTGCTGGGTTGA |
| **SjSxa2** | ATGGTAGTTTTGCACAGATTGCTTTTAAGTTTGTGGCCCGCAGGAGCCATGTTGCTAGTGTCTTCATTTCTATTGAACCTGCCAACGACATCCGCGATTCCGATTTATAGCGATCCCCACCAATGTTCACTTAGGGTTAGCGTTAGAACAGATGTCGAGGGGGAGCAACAACAGACTCAGGCAATGGGCTCTGTCGTTATTGCGTCAAATGTAACGTCATCCGACGCGCCGACCGAAAACCCCACGTCTAGTGCGCCAACGACTTTTAGTCACACTGTTAACAGTACATTTCCTACCCATACGCCAACGACCAGTACCTCTACAAATACGCCCACGGACGCAAGAGTCAGCTCTTTACCTTTATTGAACGGCGACATGCCCGCACTGTATGCCGGCTACCTTAACGTAAGCGCAAGCTCAACGTCCCCACATTCTATTTTCTACGCGTATGCACCTTCTGAACAAGGGTCCGATGATTTGATCGTATGGCTGCAAGGTGGGCCGGGATGCGCAGGCACTATTGGACTTTTCGCCGAAAATGGGCCGGTACGTTTCGGTCCTGGCGACAGCGCTCCCGTTCGTTCAGAACACTCTTGGACTAGATACGCAGATGTACTTTATTTGGACCAACCATTCGGGACGGGATTTTCTTACGCTGTCACCGAAGCTGACTATACGACCACAATGGATGCGGCGTCTGCAGAATTCATAGCATTTTTGGACGGATTCTTCGACGCATACCCAGATACAGCTTCTAAGAGGCTATTTTTCGTGGGGGAGTCTTACGGAAGTTTTTGGGGGGCCTACTTTGCCAGGGCCCTGGCAGATAGACCTGACATTTATGATCGTATAGTTAGTGTGGGCATCGTGTCTGGACTATCAACTACTCACGATACTCAGTACAGCGTATCTAACTCCTTATGGTTGTCTCACTTAAATAAGCTAGGCTATTATACCCAGGATAAAAGCACCTGGAATTCTTTTAGTAGAGTCAATGCACGTTGCGGTTACGACAAGGTAATGCAGGAGTTCACTTTCCCAGCGAGTCAGACGCCGCTTAGTAGTATATCTACGAGCACCTCAAGCTCAAGTACCAGTTCTGGAGCTGGAGCCGGAGCAACGGTTTCTTCCTTGACCGATTCTTCAGGGAATGCAAGTTACACCAATTCTTCTAGCAGCGCTGAAGTAGACGTGTTTTCATACTTCCCCTCATGCGACACAAACGACGCAACGTACATGCTAATGTATTTGCAGAATACGTGTTTAATTTCTTATGACGTCTCTATCGACTGTACAACTGCTTCCCAACTTACAGATCACTTAACGCCATACTTGAATAGGGCAGACGTTAGGGAGGCCTTGCATGTCCCCTCCTCTGTGGCTGCATTTAGCACGGCAAACGGTGTGTTTGGAGACGGCTGCAACTATGATTTACTGCAGAAAATTAATACAGTTACCTACGAACCAGTTCTGAAATCAGTCGTACCGAGCTTGATCGCCGATGGTAAAAGGGTGGCCTTTCTGTCAGGCGCATTGGACTATCAGATTCCGTGGAGCGGAACCCTTTTGGCGATGCAAAATACTACTTGGGCCGGTTGGCAAGGATTTCAGACGGAGCCTGACTGGGACAACCCCGTAGGCTTTACGCTAAACGAAAGAGGATTGAGTTTTACCTTACTTAATTCTGTGGGACACATGGCCTCAATGAATGATCCAGACAGGGTGAACCGTTGGTTGCAGGAACAAGTCCTTTGA |

**Table S8.** Fungal pheromone peptides.

| **Species (peptide)** | **Amino acid sequence** |
| --- | --- |
| *S. cerevisiae* (ScPep)  [6] | WHWLQLKPGQPMY |
| *C. albicans* (CaPep) [6] | GFRLTNFGYFEPG |
| *S. pombe* (SpPep) [6] | TYADFLRAYQSWNTFVNPDRPNL |
| *S. octosporus* (SoPep) [6] | TYEDFLRVYKNWWSFQNPDRPDL |
| *S. japonicus* (SjPep) [6] | VSDRVKQMLSHWWNFRNPDTANL |

**Table S9.** Best-fit ± standard error Hill function parameters from ScSte2 alanine scan.

| Name | Peptide sequence | Bottom (A.U.) | Top (A.U.) | LogEC_50_ (M) | Hill Slope |
| --- | --- | --- | --- | --- | --- |
| ScSte2 | | | | | |
| ScPep | WHWLQLKPGQPMY | 6.8±3.0 | 380±10 | -7.2±0.1 | 2.0±0.1 |
| ScPep1A | AHWLQLKPGQPMY | 6.4±3.4 | 320±10 | -7.4±0.1 | 3.4±0.8 |
| ScPep2A | WAWLQLKPGQPMY | 4.9±2.1 | 270±10 | -7.1±0.1 | 3.7±0.2 |
| ScPep3A | WHALQLKPGQPMY | 4.2±1.6 | 280±10 | -7.2±0.1 | 2.5±0.1 |
| ScPep4A | WHWAQLKPGQPMY | 4.6±2.0 | 250±10 | -7.3±124.4 | N/A |
| ScPep5A | WHWLALKPGQPMY | 5.0±2.3 | 380±10 | -7.3±0.1 | 3.4±0.3 |
| ScPep6A | WHWLQAKPGQPMY | 3.9±2.3 | 310±10 | -7.1±0.1 | 5.6±0.7 |
| ScPep7A | WHWLQLAPGQPMY | 10±4 | 400±10 | -6.9±0.1 | 2.6±0.4 |
| ScPep8A | WHWLQLKAGQPMY | 4.5±1.7 | 430±10 | -7.1±0.1 | 2.7±0.1 |
| ScPep9A | WHWLQLKPAQPMY | 3.9±2.1 | 370±10 | -6.8±0.1 | 5.0±1.3 |
| ScPep10A | WHWLQLKPGAPMY | 4.9±2.4 | 380±10 | -6.9±0.1 | 5.3±3.7 |
| ScPep11A | WHWLQLKPGQAMY | 5.3±3.7 | 410±10 | -7.3±0.1 | 4.6±1.3 |
| ScPep12A | WHWLQLKPGQPAY | 5.9±4.7 | 410±10 | -7.3±125.9 | N/A |
| ScPep13A | WHWLQLKPGQPMA | 4.9±2.5 | 360±10 | -7.2±0.1 | 8.2±3.1 |
| ScSte2 + ScBar1 | | | | | |
| ScPep | WHWLQLKPGQPMY | 5.3±2.4 | 390±10 | -6.1±0.1 | 1.7±0.1 |
| ScPep1A | AHWLQLKPGQPMY | -3.1±5.8 | 350±10 | -5.9±0.1 | 0.76±0.06 |
| ScPep2A | WAWLQLKPGQPMY | 5.9±3.0 | 350±10 | -5.0±0.1 | 1.5±0.1 |
| ScPep3A | WHALQLKPGQPMY | 6.4±2.6 | 370±20 | -4.6±0.1 | 0.74±0.05 |
| ScPep4A | WHWAQLKPGQPMY | -1.6±2.9 | 340±10 | -5.4±0.1 | 0.72±0.04 |
| ScPep5A | WHWLALKPGQPMY | 0.52±8.7 | 340±10 | -6.3±0.1 | 1.2±0.2 |
| ScPep6A | WHWLQAKPGQPMY | 0.38±5.71 | 390±10 | -5.5±0.1 | 0.99±0.10 |
| ScPep7A | WHWLQLAPGQPMY | 5.7±1.3 | 380±10 | -5.4±0.1 | 1.7±0.1 |
| ScPep8A | WHWLQLKAGQPMY | 4.6±2.6 | 440±10 | -5.6±0.1 | 1.1±0.1 |
| ScPep9A | WHWLQLKPAQPMY | 3.7±2.1 | 440±10 | -4.8±0.2 | 1.2±0.1 |
| ScPep10A | WHWLQLKPGAPMY | 8.3±1.6 | 420±10 | -5.2±0.1 | 1.8±0.1 |
| ScPep11A | WHWLQLKPGQAMY | 8.0±2.5 | 410±10 | -5.6±0.1 | 1.5±0.1 |
| ScPep12A | WHWLQLKPGQPAY | 2.5±2.9 | 450±10 | -6.0±0.1 | 1.3±0.1 |
| ScPep13A | WHWLQLKPGQPMA | 4.6±2.5 | 490±10 | -5.1±0.1 | 1.2±0.1 |

**Table S10.** Best-fit ± standard error Hill function parameters from CaSte2 alanine scan.

| Name | Peptide sequence | Bottom (A.U.) | Top (A.U.) | LogEC_50_ (M) | Hill Slope |
| --- | --- | --- | --- | --- | --- |
| CaSte2 | | | | | |
| CaPep | GFRLTNFGYFEPG | -1.9±10 | 460±10 | -7.2±0.1 | 1.3±0.2 |
| CaPep1A | AFRLTNFGYFEPG | 14±7 | 460±9 | -7.1±0.1 | 3.0±0.4 |
| CaPep2A | GARLTNFGYFEPG | 0.97±10 | 460±20 | -7.4±0.1 | 1.4±0.3 |
| CaPep3A | GFALTNFGYFEPG | 5.7±3.4 | 460±10 | -6.9±0.1 | 2.0±0.1 |
| CaPep4A | GFRATNFGYFEPG | 3.4±4.9 | 460±10 | -6.5±0.1 | 1.3±0.1 |
| CaPep5A | GFRLANFGYFEPG | 3.8±0.3 | 15±0.5 | -5.2±0.1 | 2.7±0.7 |
| CaPep6A | GFRLTAFGYFEPG | 4.7±2.8 | 570±10 | -6.2±0.1 | 4.4±0.4 |
| CaPep7A | GFRLTNAGYFEPG | 3.4±0.2 | 34±0.4 | -5.5±0.1 | 1.4±0.1 |
| CaPep8A | GFRLTNFAYFEPG | 15.3±21 | 640±20 | -6.4±0.1 | 1.3±0.3 |
| CaPep9A | GFRLTNFGAFEPG | 17±6 | 700±10 | -6.6±0.1 | 1.9±0.1 |
| CaPep10A | GFRLTNFGYAEPG | 5.6±1.9 | 180±10 | -4.6±0.1 | 2.8±0.5 |
| CaPep11A | GFRLTNFGYFAPG | 6.1±3.9 | 180±10 | -6.5±120 | N/A |
| CaPep12A | GFRLTNFGYFEAG | -1.7±3.4 | 580±10 | -5.3±0.1 | 0.95±0.04 |
| CaPep13A | GFRLTNFGYFEPA | -4.4±8.1 | 820±10 | -7.1±0.1 | 0.81±0.04 |
| CaPep9A10A | GFRLTNFGAAEPG | 2.9±2.2 | 570±10 | -4.7±0.1 | 0.99±0.04 |
| CaPep12A13A | GFRLTNFGYFEAA | 4.9±0.5 | 170±10 | -4.8±0.1 | 2.8±0.1 |
| CaPep2A13A | GARLTNFGYFEPA | 1.5±6.2 | 790±10 | -7.0±0.1 | 1.2±0.1 |
| CaSte2 + CaBar1 | | | | | |
| CaPep | GFRLTNFGYFEPG | 5.6±17.4 | 420±20 | -6.3±0.1 | 0.87±0.17 |
| CaPep1A | AFRLTNFGYFEPG | 4.5±14.4 | 410±10 | -6.2±0.1 | 1.1±0.22 |
| CaPep2A | GARLTNFGYFEPG | 12±13 | 430±10 | -6.9±0.1 | 1.6±0.3 |
| CaPep3A | GFALTNFGYFEPG | 14±11 | 460±10 | -6.1±0.1 | 0.74±0.08 |
| CaPep4A | GFRATNFGYFEPG | 11±8 | 410±8.4 | -6.1±0.1 | 2.4±0.4 |
| CaPep5A | GFRLANFGYFEPG | 6.4±0.2 | 13±1 | -4.9±0.1 | 3.3±0.1.3 |
| CaPep6A | GFRLTAFGYFEPG | 0.83±6.2 | 450±10 | -5.9±0.1 | 1.7±0.2 |
| CaPep7A | GFRLTNAGYFEPG | 5.1±0.2 | 17±1 | -5.6±0.1 | 2.2±0.4 |
| CaPep8A | GFRLTNFAYFEPG | 13±8 | 460±10 | -5.5±0.1 | 1.0±0.1 |
| CaPep9A | GFRLTNFGAFEPG | 10±7 | 480±10 | -5.9±0.1 | 1.7±0.2 |
| CaPep10A | GFRLTNFGYAEPG | 6.0±0.4 | 98±1 | -4.2±0.1 | 2.6±0.13 |
| CaPep11A | GFRLTNFGYFAPG | 5.4±2.2 | 120±10 | -5.8±0.1 | 1.5±0.2 |
| CaPep12A | GFRLTNFGYFEAG | 2.2±4.8 | 540±10 | -5.0±0.1 | 1.1±0.1 |
| CaPep13A | GFRLTNFGYFEPA | 14±6 | 590±10 | -5.7±0.1 | 0.94±0.06 |
| CaPep9A10A | GFRLTNFGAAEPG | 3.5±3.8 | 510±20 | -4.6±0.1 | 1.0±0.1 |
| CaPep12A13A | GFRLTNFGYFEAA | 5.6±0.5 | 11-0±10 | -4.5±0.1 | 1.8±0.1 |
| CaPep2A13A | GARLTNFGYFEPA | -2.4±22.85 | 680±20 | -6.5±0.1 | 0.86±0.10 |

**References**

[1] A. M. William Shaw, H. Yamauchi, J. Mead, M. Wigglesworth, G. Ladds, T. Ellis Correspondence, W. M. Shaw, G.-O. F. Gowers, D. J. Bell, N. Larsson, and T. Ellis, *Engineering a Model Cell for Rational Tuning of GPCR Signaling*, Cell **177**, 1 (2019).

[2] M. Jin, B. Errede, M. Behar, W. Mather, S. Nayak, J. Hasty, H. G. Dohlman, and T. C. Elston, *Yeast Dynamically Modify Their Environment to Achieve Better Mating Efficiency*, Sci. Signal. **4**, ra54 (2011).

[3] S. K. Jones, S. C. Clarke, C. S. Craik, and R. J. Bennett, *Evolutionary Selection on Barrier Activity: Bar1 Is an Aspartyl Protease with Novel Substrate Specificity*, Am. Soc. Microbiol. **6**, e01604 (2015).

[4] G. Ladds and J. Davey, *Sxa2 Is a Serine Carboxypeptidase That Degrades Extracellular P-Factor in the Fission Yeast Schizosaccharomyces Pombe*, Mol. Microbiol. **36**, 377 (2000).

[5] A. C. Burkholder and L. H. Hartwell, *The Yeast α-Factor Receptor: Structural Properties Deduced from the Sequence of the STE2 Gene*, Nucleic Acids Res. **13**, 8463 (1985).

[6] S. Billerbeck, J. Brisbois, N. Agmon, M. Jimenez, J. Temple, M. Shen, J. D. Boeke, and V. W. Cornish, *A Scalable Peptide-GPCR Language for Engineering Multicellular Communication*, Nat. Commun. **9**, 1 (2018).
